# Inhibition of monocyte-like cell extravasation protects from neurodegeneration in DBA/2J glaucoma

**DOI:** 10.1186/s13024-018-0303-3

**Published:** 2019-01-22

**Authors:** Pete A. Williams, Catherine E. Braine, Krishnakumar Kizhatil, Nicole E. Foxworth, Nicholas G. Tolman, Jeffrey M. Harder, Rebecca A. Scott, Gregory L. Sousa, Alyssa Panitch, Gareth R. Howell, Simon W. M. John

**Affiliations:** 1 The Jackson Laboratory, Bar Harbor, ME USA; 2 Department of Clinical Neuroscience, Section of Ophthalmology and Vision, St. Erik Eye Hospital, Karolinska Institutet, Stockholm, Sweden; 3 Department of Materials Science and Engineering, University of Delaware, Newark, DE USA; 4 Department of Biomedical Engineering, University of California, Davis, CA USA; 5 Graduate Program in Genetics, Sackler School of Graduate Biomedical Sciences, Tufts University, Boston, MA USA; 6 Department of Ophthalmology, Tufts University of Medicine, Boston, MA USA; 7 The Howard Hughes Medical Institute, Bar Harbor, ME USA

**Keywords:** Glaucoma, Retinal ganglion cell, Optic nerve, Monocyte, Vascular leakage, Extravasation, Platelet, Neuroinflammation, RNA-sequencing

## Abstract

**Background:**

Glaucoma is characterized by the progressive dysfunction and loss of retinal ganglion cells. Recent work in animal models suggests that a critical neuroinflammatory event damages retinal ganglion cell axons in the optic nerve head during ocular hypertensive injury. We previously demonstrated that monocyte-like cells enter the optic nerve head in an ocular hypertensive mouse model of glaucoma (DBA/2 J), but their roles, if any, in mediating axon damage remain unclear.

**Methods:**

To understand the function of these infiltrating monocyte-like cells, we used RNA-sequencing to profile their transcriptomes. Based on their pro-inflammatory molecular signatures, we hypothesized and confirmed that monocyte-platelet interactions occur in glaucomatous tissue. Furthermore, to test monocyte function we used two approaches to inhibit their entry into the optic nerve head: (1) treatment with DS-SILY, a peptidoglycan that acts as a barrier to platelet adhesion to the vessel wall and to monocytes, and (2) genetic targeting of *Itgam* (CD11b, an immune cell receptor that enables immune cell extravasation).

**Results:**

Monocyte specific RNA-sequencing identified novel neuroinflammatory pathways early in glaucoma pathogenesis. Targeting these processes pharmacologically (DS-SILY) or genetically (*Itgam* / CD11b knockout) reduced monocyte entry and provided neuroprotection in DBA/2 J eyes.

**Conclusions:**

These data demonstrate a key role of monocyte-like cell extravasation in glaucoma and demonstrate that modulating neuroinflammatory processes can significantly lessen optic nerve injury.

**Electronic supplementary material:**

The online version of this article (10.1186/s13024-018-0303-3) contains supplementary material, which is available to authorized users.

## Background

Glaucoma is the leading cause of irreversible blindness, affecting ~ 70 million people worldwide [1, 2]. It is a complex, multifactorial disease characterized by the progressive dysfunction and loss of retinal ganglion cells (RGCs). Age, genetics, and intraocular pressure (IOP) are all major risk factors.

Neuroinflammation may be a critical, and treatable, pathogenic event in glaucoma [3]. Although a number of studies have examined neuroinflammatory pathways in animal models of glaucoma, the molecular identities of the cell types involved and how these cells initiate damage need further elucidation. Recent work has demonstrated expansion of CD163^+^ macrophage populations in the optic nerves of human glaucoma patients [4], however the role of infiltrating immune cells in glaucoma pathogenesis is unknown. Interestingly, targeting neuroinflammatory pathways in multiple models of glaucoma has been protective [3, 5–14]. As glaucoma shares many neurodegenerative / neuroinflammatory events with other common neurodegenerations [15, 16] understanding the molecular identities and roles of these damaging cells is of paramount importance in developing novel therapeutic strategies for diseases in which neuroinflammation may play a key role.

Accumulating data suggest that key molecular events driving RGC degeneration following a chronic increase in IOP happen within the optic nerve head (ONH) where RGC axons exit the eye [3, 5, 17–19]. At this location the axons are clustered into bundles and remain unmyelinated. In the mouse, the blood supply of the unmyelinated ONH is in direct contact with neurons and forms a neuro-glial-vascular complex of pericytes, vascular endothelial cells, astrocytes, and retinal ganglion cell axons [8, 20]. The ONH contains a rich neuro-vascular network, and thus may be particularly sensitive to neuroinflammatory insults.

The DBA/2 J mouse develops glaucoma with the hallmark features of an inherited, chronic human glaucoma [21, 22]. In our colony, elevated IOP develops from 6 months of age onwards and IOP has increased in the majority of eyes by 9 months of age. Axon degeneration in the optic nerve is present by 10.5 months of age, while the majority of eyes have severe glaucomatous neurodegeneration by 12 months of age. During this glaucomatous disease progression, a specific class of monocyte-like cells exits the vasculature and enters the ONH (CD45^hi^/CD11b^+^/CD11c^+^) [18]. To date one of the most protective strategies in DBA/2 J glaucoma is a radiation therapy [23]. In this strategy, a sub-lethal dose of γ-radiation or a local (eye-only) dose of X-ray radiation at 2–3 months of age profoundly protects RGCs (~ 96% of eyes have no glaucoma at 12 months of age) [18]. Most notably, this radiation therapy prevents the infiltration of the monocyte-like cell population. Therefore, strategies to prevent monocyte infiltration into the ONH are expected to have therapeutic value. However, previous studies have not adequately tested the role of cellular infiltration, whether these monocyte-like cells are responsible for damage or are simply bystanders remains to be elucidated.

Using microarray analysis of whole ONHs in DBA/2 J mice following radiation therapy we identified *Glycam1* as a candidate molecule to mediate protection [18]. Supporting this, genetic knockout of *Glycam1* on a DBA/2 J background increased glaucoma susceptibility (i.e. increased the risk that an eye would develop severe glaucoma) following radiation therapy [24]. Although genetic ablation of *Glycam1* restored entry of monocyte-like cells into the ONH, glaucoma susceptibility was more modestly affected [24]. The reasons for this require further evaluation and may reflect the complex, context dependent regulation of both cellular recruitment and cellular phenotypes following entry into the ONH. This highlights the importance of understanding the roles and molecular identity of these monocytes in glaucoma.

Here, we use RNA-sequencing to characterize ONH monocyte-like cell populations and identify novel inflammatory pathways in early glaucoma following periods of elevated IOP. We identify key pathways pertaining to monocyte-like entry including PDGF signalling and monocyte-platelet binding. We then show that preventing monocyte-like cell extravasation using the peptidoglycan DS-SILY provides a period of optic nerve protection during glaucoma pathogenesis in DBA/2 J mice. Following these experiments, we genetically ablate *Itgam* (CD11b, an important cell adhesion molecule in extravasation and a platelet-fibrinogen receptor). This prevents monocyte-like cell entry and limits glaucoma pathogenesis, thus identifying CD11b as a key neuroinflammatory molecule. Taken together, our data support a model whereby monocyte-like cell entry is pathogenically important in DBA/2 J glaucoma. These data suggest that therapeutic strategies that target these cells will have therapeutic value in glaucoma and possibly an array of other neuroinflammatory conditions.

## Materials and methods

### Mouse strain, breeding and husbandry

Mice were housed and fed in a 14 h light / 10 h dark cycle with food and water available ad libitum [9]. All breeding and experimental procedures were undertaken in accordance with the Association for Research for Vision and Ophthalmology Statement for the Use of Animals in Ophthalmic and Research. The Institutional Biosafety Committee (IBC) and the Animal Care and Use Committee (ACUC) at The Jackson Laboratory approved this study. C57BL/6 J (B6), DBA/2 J (D2) and D2-*Gpnmb*^*+*^ strains were utilized and have been described in detail elsewhere [25]. In DBA/2 J mice, mutations in two genes (*Gpnmb*^*R150X*^ and *Tyrp1*^*b*^) drive an iris disease with features of human iris atrophy and pigment dispersion syndrome [22, 25–29]. In this disease, pigment disperses from the iris and induces damage in the drainage structures of the eye. This inhibits aqueous humour outflow and leads to an increase in IOP. We used D2-*Gpnmb*^*+*^ mice as a control, a non-glaucomatous substrain of DBA/2 J that does not develop elevated IOP [22]. D2.129S4(B6)-*Itgam*^*tm1Myd*^/Sj mice (D2.*Itgam*^*−/−*^; JR# 31084) were generated by backcrossing B6.129S4-*Itgam*^*tm1Myd*^/J mice (JR# 003991), heterozygous for the *Itgam*^*tm1Myd*^ allele, to DBA/2 J a minimum of ten times (>N10) before intercrossing to generate mice homozygous for the *Itgam*^*tm1Myd*^ allele (>N10F1). The presence of the allele was confirmed by standard PCR genotyping.

### γ-Radiation therapy

A sub-lethal dose of γ-radiation (7.5Gy) was administered using a ^137^Cesium source in a single dose at 10–12 weeks of age. Mice were placed on a rotating platform to ensure uniform administration of the treatment. Mice were monitored follow radiation treatment. In our colony this level of treatment does not result in any adverse conditions and mice do not require bone marrow reconstitution [18].

### Clinical examination

D2 mice develop elevated intraocular pressure and glaucoma subsequent to an iris disease. In all D2 glaucoma experiments, the progression of the iris disease and intraocular pressure in mutant or drug-treated mice were compared to control D2 mice using previously described methods [30]. In each experiment, iris disease and intraocular pressure were assessed in 40 eyes per genotype or treatment. Iris disease was assessed at two-month intervals starting at 6 months of age until experiment completion. Intraocular pressure was measured at 45-day intervals beginning at 8.5–9 months of age until experiment completion.

### DS-SILY and LPS administration

D2 or B6 mice were injected with 1–100 μM DS-SILY or DS-SILY_BIOTIN_ [31, 32] in sterile saline once every 7–21 days by intravenous, intraperitoneal, or subcutaneous routes with or without ~ 1 μg/g[body weight] LPS in sterile saline intraperitoneally (LPS O111:B4, Sigma). Initial route and dose testing experiments were performed in 10 week old mice. For glaucoma neurodegeneration experiments mice were injected intravenously once every 21 days from 6 to 7 to 10.5 or 12 months of age with 25 μM DS-SILY_BIOTIN_ in sterile saline.

### FAC sorting

FAC sorting was performed as previously described with some adjustments [33]. Prior to cell collection, all surfaces and volumes were cleaned with 70% ethanol and RNaseZap (ThermoFisher Scientific) solution followed by dH_2_0. Mice were euthanized by cervical dislocation, eyes enucleated and placed immediately into ice-cold HBSS. Single ONHs were dissected from the eyes in HBSS on ice and placed directly into 100 μl of a custom HBSS (Gibco), dispase (5 U/ml) (Stemcell Technologies), DNase I (2000 U/ml) (Worthington Biochemical) and SUPERase (1 U/μl) (ThermoFisher Scientific) solution. Samples were incubated for 20 mins at 37 °C and shaken at 350 RPM in an Eppendorf Thermomixer R followed by gentle trituration using a 200 μl pipette. For PBMC samples whole blood was collect into EDTA saline via cheek bleed in live mice just prior to euthanizing for ONH collection. Blood samples were centrifuged, EDTA saline removed, and then replaced with ACK buffer on ice for 5 min. Following this, blood samples were centrifuged, ACK buffer was removed, and the following protocol was followed for both ONH and PBMC samples. There is a caveat as we are forced to assess PBMCs from a different tissue as there is no corresponding control for monocyte-like cells in the glaucomatous ONH (i.e. there are no ONH monocytes in genetic or treated controls, Additional file 1: Figure S1). Samples were blocked in 2% BSA, SUPERase (1 U/μl) in HBSS, and stained with antibodies secondary conjugated antibodies against CD11b, CD11c, CD34, CD45.2, GFAP, as well as DAPI. This cocktail allowed other retina cell types to be accurately removed during FACS. FACS was performed on a FACSAria (BD Biosciences) and CD45.2^hi^/CD11b^+^/CD11c^+^ (and negative for all other markers) monocyte-like cells were sorted into 100 μl buffer RLT + 1% β-ME, vortexed and frozen at − 80 °C until further processing.

### RNA-sequencing and analysis

Monocytes from single optic nerve heads or from peripheral blood (restrained cheek bleed) were FAC sorted into 100 μl buffer RLT + 1% βME and frozen at − 80 °C until further processing. Samples were defrosted on ice and homogenized by syringe in RLT Buffer (total volume 300 μl). Total RNA was isolated using RNeasy micro kits as according to manufacturer’s protocols (Qiagen) including the optional DNase treatment step, and quality was assessed using an Agilent 2100 Bioanalyzer. The concentration was determined using a Ribogreen Assay from Invitrogen. Amplified dscDNA libraries were created using a Nugen Ovation RNA-seq System V2 and a primer titration was performed to remove primer dimers from the sample to allow sample inputs as low as 50 pg RNA. The SPIA dscDNA was sheared to 300 bp in length using a Diogenode Disruptor. Quality control was performed using an Agilent 2100 Bioanalyzer and a DNA 1000 chip assay. Library size produced was analysed using qPCR using the Library Quantitation kit/Illumina GA /ABI Prism (Kapa Biosystems). Libraries were barcoded, pooled, and sequenced 6 samples per lane on a HiSeq 2000 sequencer (Illumina) giving a depth of 30–35 million reads per sample.

Following RNA-sequencing samples were subjected to quality control analysis by a custom quality control python script. Reads with 70% of their bases having a base quality score ≥ 30 were retained for further analysis. Read alignment was performed using TopHat v 2.0.7 [34] and expression estimation was performed using HTSeq [35] with supplied annotations and default parameters against the DBA/2 J mouse genome (build-mm10). Bamtools v 1.0.2 [36] were used to calculate the mapping statistics. Differential gene expression analysis between groups was performed using edgeR v 3.10.5 [37] following, batch correction using RUVSeq, the removal of outlier samples and lowly expressed genes by removing genes with less than five reads in more than two samples. Normalization was performed using the trimmed mean of M values (TMM). Unsupervised HC was performed in R (1-cor, Spearman’s *rho*). Following preliminary analysis, 1 sample was removed as an outlier. Adjustment for multiple testing was performed using false discovery rate (FDR). Genes were considered to be significantly differentially expression at a false discovery rate (FDR; *q*) of *q* < 0.05. Pathway analysis was performed in R, IPA (Ingenuity Pathway Analysis, Qiagen), and using publically available tools (see Results).

### Axon labelling with PPD and grading of glaucomatous damage

The processing of optic nerves and staining with paraphenylenediamine (PPD) was as published [38]. PPD stains the myelin sheath of all axons but darkly stains the axoplasm of only damaged axons. It is well established to provide a very sensitive measure of optic nerve damage. Briefly, intracranial portions of optic nerves were fixed in 4% PFA at RT for 48 h, processed and embedded in plastic. A segment of optic nerve from within a region up to 1 mm from the posterior surface of the sclera was sectioned (1 μm thick sections) and stained with PPD. Typically 30–50 sections are taken from each nerve. Multiple sections of each nerve were considered when determining damage level. Optic nerves were analyzed and determined to have one of 3 damage levels:

1. No or early damage (NOE) – less than 5% axons damaged and no gliosis. This level of damage is seen in age and sex matched non-glaucomatous mice and is not due to glaucoma. Although none of these eyes exhibit glaucomatous nerve damage, this damage level is called no or early glaucoma as some of these eyes have early molecular changes that precede neurodegeneration. These molecular changes can be detected by gene expression studies [9]. Eyes with these early molecular changes but no degeneration are considered to have early glaucoma when discussing metabolic, mitochondrial and gene expression changes in this paper.
2. Moderate damage (MOD) – average of 30% axon loss and early gliosis,
3. Severe (SEV) – > 50% axonal loss and damage with prominent gliosis.

Irrespective of treatment group, eyes with any degree of optic nerve severity have been demonstrated to have similar distributions of axon and soma loss. Importantly, eyes with NOE graded optic nerves are indistinguishable from controls (D2-*Gpnmb*^*+*^) in terms of axon and soma numbers [5, 33]. The only exception is when somal and axonal degeneration pathways are uncoupled in mice with mutations that prevent somal but not axonal degeneration (in these cases mice with SEV graded optic nerves have not lost their retinal ganglion cell somas) [39]. Throughout this manuscript to clarify that there is no axon/soma disease uncoupling we perform retinal ganglion cell soma counts on retinas with corresponding NOE and SEV graded optic nerves.

### Flow cytometry

Mice were euthanized by cervical dislocation, eyes enucleated, and placed immediately into ice-cold HBSS. Optic nerve heads were dissected from eyes in HBSS on ice and placed directly into a dispase and DNase solution. Samples were incubated for 30 mins at 37 °C and shaken at 350 RPM. Following this samples were blocked in 2% BSA in HBSS and stained with antibodies against CD11b (PE or PE-Cy7), CD11c (FITC, PE, or APC), CD45.2 (AF647 or BV), and/or CD41 (FITC). Samples were stained with PI for 5 mins before being run on the flow cytometer. Four-colour flow cytometry was performed on a FACSCaliber (BD Bioscience) or imaging cytometry on an Amnis ImageStream X. Samples were run to completion. Cell populations were analysed using FloJo.

### Immunofluorescent staining of retinal whole mounts and sections

Mice were euthanized by cervical dislocation, their eyes enucleated and placed in 4% PFA ON. Retinas were dissected and flatmounted onto slides, permeabilized with 0.1% Triton-X for 15 mins, blocked with 2% BSA in PBS and stained ON at RT in primary antibody (1:500 anti-RBMPS; Novus Biologicals, NB100–105). After primary antibody incubation, retinas were washed 5 times in PBS, stained for 4 h at RT with secondary antibody (AF594 or AF488). Slides were then washed a further 5 times with PBS, stained with DAPI for 15 mins, mounted with fluoromount, coverslipped and sealed with nail-polish. For retinal sections, eyes were cryoprotected in 30% sucrose ON, frozen in OCT and cryosectioned at 18 μm. Slides were warmed to room temperature and the procedure above was followed. For immunofluorescence against platelets, pre-conjugated antibodies (as in *Flow Cytometry*) were used. Retinas were imaged on a Zeiss Axio Observer for low resolution counts. Retinal sections were imaged on a Leica SP5 for higher resolution images.

### Nissl staining of frozen retinal sections

Mice were euthanized by cervical dislocation, their eyes enucleated and placed in 4% PFA ON. Following this eyes were cryoprotected in 30% sucrose, frozen in OCT and cryosectioned at 18 μm. Slides were warmed to room temperature, placed in 1:1 alchol:chloroform ON, and rehydrated through serial alcohol gradient. Slides were washed once in distilled water and stained for 15 mins in 0.1% cresyl violet in distilled water before being differentiated in 95% alcohol, dehydrated in 100% alcohol and cleared in xylene. Slides were left to dry at RT, mounted with fluoromount, coverslipped and sealed with nail-polish. Sections were imaged using a Nikon Eclipse E200.

### Assessment of vascular permeability

To assess retinal vascular permeability mice were restrained and intravenously tail vein injected with 0.02% Hoechst 33342 in sterile saline. Fifteen minutes after injection, mice were euthanized by cervical dislocation and eyes enucleated and placed in 4% PFA for 2 h. Retinas were subsequently dissected, flatmounted onto slides and imaged as above. Eight representative images per retina were taken, and the number of Hoechst+ nuclei in the ganglion cell layer (excluding vascular endothelial cells) were counted.

### Statistical analysis

The sample size (number of eyes, *n*) is shown in each figure legend. Graphing and statistical analysis was performed in R. *Student’s t* test was used for pairwise analysis in quantitative plots and error bars refer to standard error of the mean unless otherwise stated. For nerve grade comparisons *Fisher’s exact* test was used. * = *P* < 0.05, ** = *P* < 0.01, *** = *P* < 0.001.

## Results

### Monocyte specific RNA-sequencing identifies novel neuroinflammatory pathways early in glaucoma pathogenesis

To characterize monocyte-like cells in the ONH following periods of elevated IOP, we first analysed ONHs from DBA/2 J (D2) mice by flow cytometry. In our colony, iris disease occurs at ~ 6 months of age with elevated IOP having been present in the majority of eyes by 9 months of age. At this 9 months of age time point there are already transcriptomic changes to RGCs, but without any overt histological disease phenotype [33]. Consistent with our previous study [18], we first demonstrate that CD45^hi^/CD11b^+^/CD11c^+^ monocytes are present in the ONHs of 9 month old D2 mice. These cells were not present in ONHs of control mice (D2-*Gpnmb*^+^; a substrain of D2 mice that do not develop elevated IOP) or radiation-treated D2 mice that are protected from glaucoma [18, 23] (Additional file 1: Figure S1). Although IOP is elevated at this time point, there is no detectable damage to the optic nerve. Together, this suggests that CD45^hi^/CD11b^+^/CD11c^+^ monocyte-like cells are part of a very early disease process.

To elucidate the mechanisms of monocyte entry and to determine the molecular changes that occur in these cells early in disease, we performed RNA-sequencing on the RNA from CD45^hi^/CD11b^+^/CD11c^+^ monocytes from the ONH and from peripheral blood of pre-neurodegenerative 9 months of age D2 mice (Additional file 2: Figure S2). We use peripheral blood monocytes (PBMCs; with the same marker profile of CD45^hi^/CD11b^+^/CD11c^+^) as a control as there is no monocyte entry in control D2-*Gpnmb*^+^ or young D2 eyes. The corresponding optic nerves of all analysed eyes were confirmed to have had no detectable glaucomatous changes (by analysing optic nerve cross sections, *data not shown*). A total of 26 samples across all groups were successfully amplified and sequenced.

**Fig. 1:**
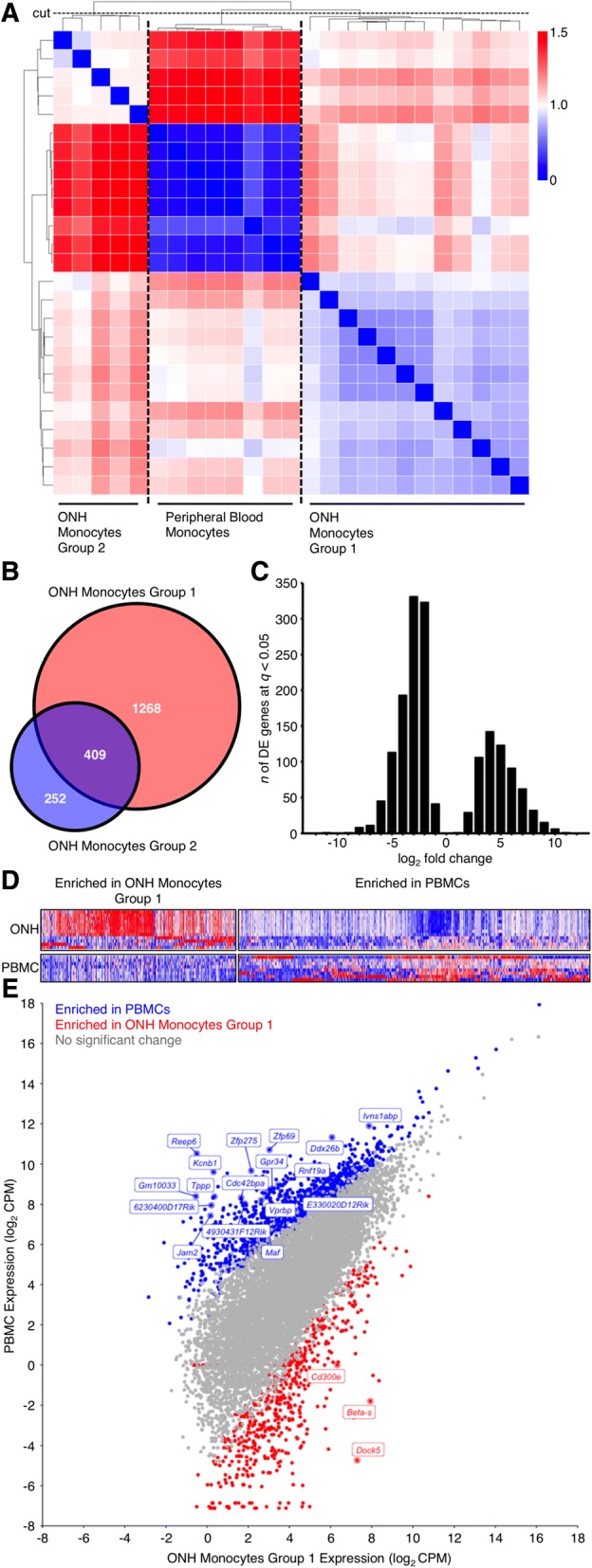
RNA-sequencing of monocytes in glaucoma. **a** Heatmap correlations of all samples (*Spearman’s rho*, *blue* = high correlation, *red* = lowest correlation). Dendrogram is shown in grey at top and left of heatmap. The cut-off to generate clusters on the dendrogram is shown as the horizontal dashed line labelled “cut”. Cutting strategy was defined as all control (peripheral blood monocytes; PBMC) samples falling into one group with the maximum number of total samples retained. **b** Venn diagram showing number of DE genes (FDR, *q* < 0.05) between ONH Monocytes Group 1 vs. PBMCs and ONH Monocytes Group 2 vs. PBMCs (*red* and *blue*), and shared DE genes between groups (*purple*). **c** Histogram of DE genes by fold change between ONH Monocytes Group 1 vs. PBMCs. **d** Heatmap showing all DE genes (*x*) by sample (*y*). *Red* = highly expressed, *blue* = lowly expressed. **e** Scatter plot showing all genes (dots, not DE = *grey*, DE and enriched in PBMCs = *blue*, DE and enriched in ONH Monocytes Group 1 = *red*). Top 20 DE genes are named

We have previously used hierarchical clustering (HC) to define molecularly distinct groups within microarray gene expression studies from whole retina and whole ONH, or following RNA-sequencing of RGC RNA [9, 33, 40]. Thus, we reasoned that HC would allow us to define molecularly distinct groups of ONH monocyte samples. Our criteria for HC groups were that all controls clustered together and that the maximum number of samples was represented. Using this strategy, HC allowed clustering of samples into 3 distinct clusters in which all peripheral blood monocyte samples clustered into one group while the ONH monocyte samples clustered into an additional 2 groups (ONH Monocytes Group 1 and 2) (Fig. 1a-e). Pairwise comparisons were made between each group.

ONH Monocytes Group 1 vs. peripheral blood monocytes had the largest number of differentially expressed (DE) genes (*n* = 1677 genes) and shared the majority of DE genes present in ONH Monocytes Group 2 vs. peripheral blood monocytes (62% of genes) (Fig. 1b, Additional file 3: Figure S3). In addition to a greater number of DE genes, samples in the Group 1 cluster appeared to have a more inflammatory phenotype as determined by a higher expression of *Itgax* [CD11c] and lower expression of *Cx3cr1* [41] (Additional file 4: Figure S4). Subsequent pathway analysis identified highly enriched inflammatory pathways in ONH monocytes including B cell and T cell signalling, chemokine and cytokine signalling pathways, cell adhesion, thrombin, and PDGF signalling pathways (Additional file 5: Table S1, Additional file 6: Figure S5).

### Alternative splicing analysis identifies additional neuroinflammatory pathways in early glaucoma pathogenesis

**Fig. 2:**
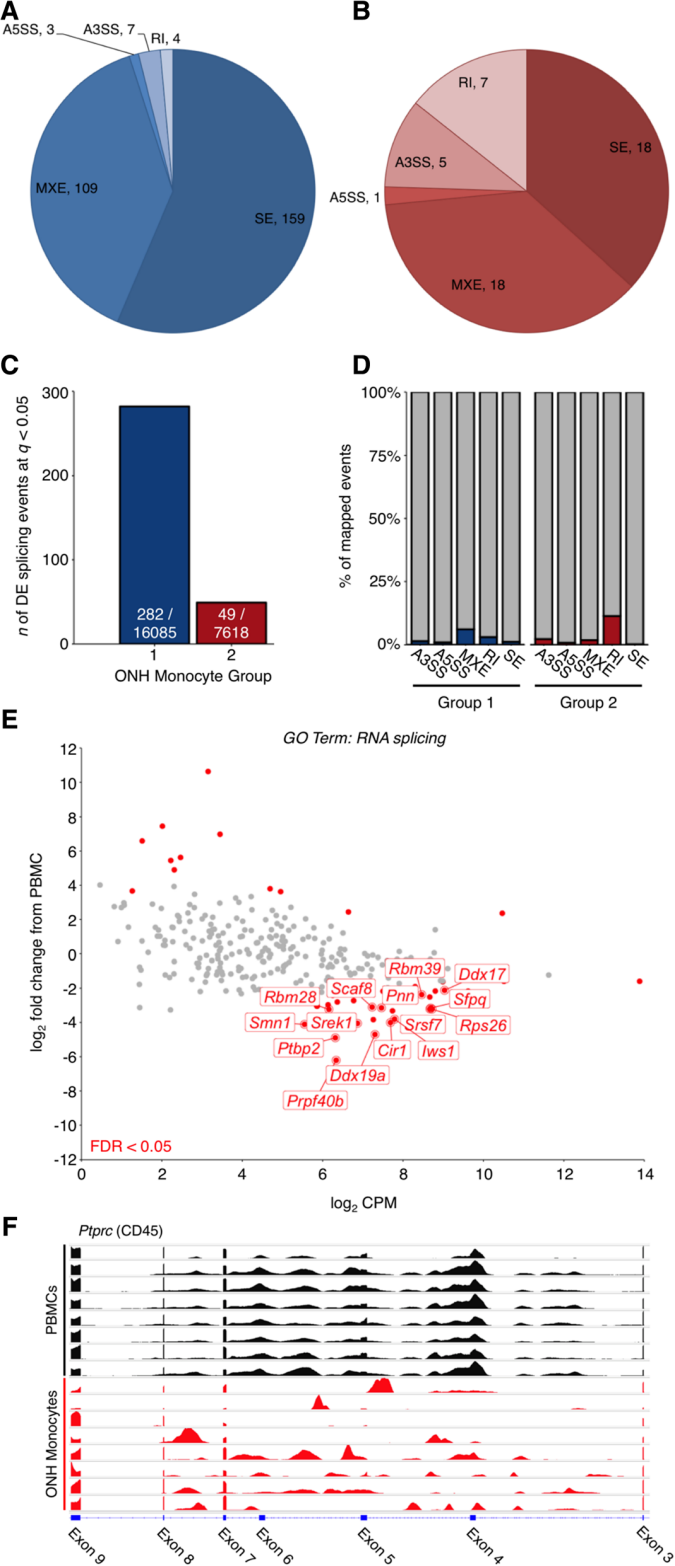
RNA alternate splicing in ONH monocytes. RNA alternative splicing analysis was performed using MATS [42]. **a** and **b** Pie diagrams showing number of differentially expressed splicing events (FDR < 0.05) between ONH Monocytes Group 1 (**a**) and ONH Monocytes Group 2 (**b**) vs. PBMCs. Events were classed as either A3SS (alternate 3’ SS), A5SS (alternate 5’ SS), RI (retained introns), SE (skipped exons), or MXE (mutually exclusive exons). **c** Total number of DE splicing events / total number of mapped splicing events. **d** Percentage of DE splicing events. **e** Scatter plot for all genes under the GO Term: RNA splicing (GO:0006395) in ONH Monocytes Group 1 vs. PBMCs. *Grey* = not DE, *red* = DE (FDR < 0.05). DE genes at FDR < 0.01 with a log_2_ CPM > 5 are named. **f** Transcriptome plot (IGV) showing alternative splicing (skipping of exons 4, 5, and 6) of *Ptprc* (CD45). *Black* = PBMC representative samples, *red* = ONH monocyte representative samples

Alternative splicing increases transcriptomic complexity by producing multiple mRNA isoforms from single genes. To assess RNA alternative splicing in our monocyte populations we utilized MATS (Multivariate Analysis of Transcript Splicing [42]) to analyse reads from ONH and peripheral monocytes in our groupings as above. We found that there were a large number of potential splicing variants in monocyte genes, and this was more enriched in Group 1 vs. PBMCs than Group 2 vs. PMBCs. MATS allowed the discovery of 5 different splice isoforms; alternate 3′ SS (splice site; A3SS), alternate 5′ SS (A5SS), retained introns (RI), skipped exons (SE), and mutually exclusive exons (MXE). Of these, SEs and MXEs were the most abundant in our data sets accounting for ~ 90% of differentially expressed splicing events in Group 1 and ~ 75% events in Group 2 (Fig. 2a-d). Further KEGG pathway analysis showed a number of DE genes associated with RNA splicing in the Group 1 vs. PBMCs dataset (GO TERM: RNA Splicing) (Fig. 2e). Pathway analysis of differentially spliced transcripts from Group 1 showed enriched pathways related to eIF2 and mTOR signalling (both implicated in glaucoma pathogenesis [33]), as well as eIF4 signalling (implicated in multiple sclerosis [43, 44]), and leukocyte extravasation signalling (Additional file 7: Table S2). eIF2 signalling is particularly interesting as; (1) eIF2 signalling regulates proinflammatory cytokine expression [45], (2) phosphorylation of eIF2α promotes translation of ATF4 mRNA which regulates genes important for resistance to oxidative stress and glutathione synthesis [46, 47]. Cytokine expression and oxidative stress have been demonstrated in glaucoma [33, 48], and (3) eIF2 signalling was the most enriched pathway in D2 RGCs compared with no glaucoma controls in our previous study [33]. This points to an interesting nexus of neuroinflammation, oxidative stress, and cytokine expression during early glaucoma pathogenesis.

We next used IGV (Integrative Genomics Viewer [49, 50]) to examine the individual genes affected by alternative splicing as certain transcript isoforms are associated with a pro-inflammatory response in immune cells [51]. Many genes that confer a pro-inflammatory status in immune cells were alternatively spliced in our datasets (Additional file 8: Table S3). One such example is *PTPRC* (CD45, *Ptprc* in mouse), a well-studied example of alternative splicing in human pro-inflammatory immune cells [51]. PTPRC / CD45 is a transmembrane protein tyrosine phosphatase that is critical for activation of homeostatic T cells [52]. Exons 4, 5, and 6 of *PTPRC* are variable cassette exons that are alternatively spliced to create five mRNA products. In activated T cells, the predominant isoform exhibits skipping of all three cassette exons [51, 52]. Interestingly, we observed skipping of *Ptprc* exons 4, 5, and 6 in Group 1 monocytes compared with PBMCs (Fig. 2f). Changes in the expression of *Ptprc* isoforms between Group 1 monocytes and PBMCs suggests a shift in transcript splicing patterns indicative of a pro-inflammatory cell output. This provides further evidence that cells enter the ONH from the periphery and become damaging, and may be an initiating factor in axon damage in the ONH during early glaucoma pathogenesis.

### RNA-sequencing implicates a role for monocyte-platelet binding in glaucoma

Due to the large number of DE genes between ONH Monocytes Group 1 and peripheral blood monocytes, we sought further definition of differentially expressed gene set regulation by generating gene modules. We assessed changes that were driving differential gene expression by generating modules of only differentially expressed genes from ONH Monocytes Group 1 vs. peripheral blood monocytes by 1-cor clustering (Spearman’s *rho*). This clustering strategy allowed the generation of 5 modules with at least 200 genes to enable pathway analysis (Modules 1–5). Initial IPA pathway analysis of gene sets from each module discovered a unique set of pathways in each module, with metabolic dysfunction and pro-inflammatory conditions being an overall theme. Module 1 was characterized by metabolic pathways especially mitochondrial dysfunction and mTOR signalling, both implicated in glaucoma [33, 53–55], Module 2 by inflammatory pathways (IL10, IL6, PI3K in macrophages), Module 3 by RNA/DNA events related to both splicing and repair, Module 4 by metabolic pathways (glycogen, cholesterol, mitochondrial dysfunction), and Module 5 by repair pathways (ATM signalling) and platelet aggregation (thrombin signalling) (Additional file 9: Figure S6, Additional file 10: Table S4).

The genes and pathways implicated in Module 5 are particularly interesting as it gave further clues to the identity of these monocyte-like cells. Thrombin (thrombin signalling –log *P* = 2.1) is a potent chemotactic pathway for macrophages [56, 57] and is mitogenic for astrocytes [58, 59]. Microglial activation and glial proliferation are implicated in glaucoma [8, 9, 60–62]. Thrombin signalling promotes clotting by converting fibrinogen to fibrin, by triggering the release of platelet activators, and triggering morphological changes to platelets [63, 64]. In addition, thrombin can directly act on the vascular endothelium promoting vasodilation and increased permeability [65, 66].

Further pathway analysis using PANTHER classification analysis (protein analysis through evolutionary relationships [67]) identified PDGF signalling as one of the top enriched pathways. Our modular analysis further implicated the role of PDGF signalling in ONH monocytes and PDGF signalling was in the top 2 enriched pathways in 3/5 of our modules (Additional file 11: Table S5). PDGF can lead to MAPK and PI3K activation, platelet activation, lymphocyte motility, in addition to being a potent chemoattractant [68–70]. Although PDGF receptors are not expressed on peripheral blood monocytes, PDGFRβ is induced upon differentiation into macrophages [70]. This lends further evidence for monocyte-like cells entering the ONH and differentiating into potentially damaging macrophage/microglial-like cells in DBA/2 J glaucoma.

**Fig. 3:**
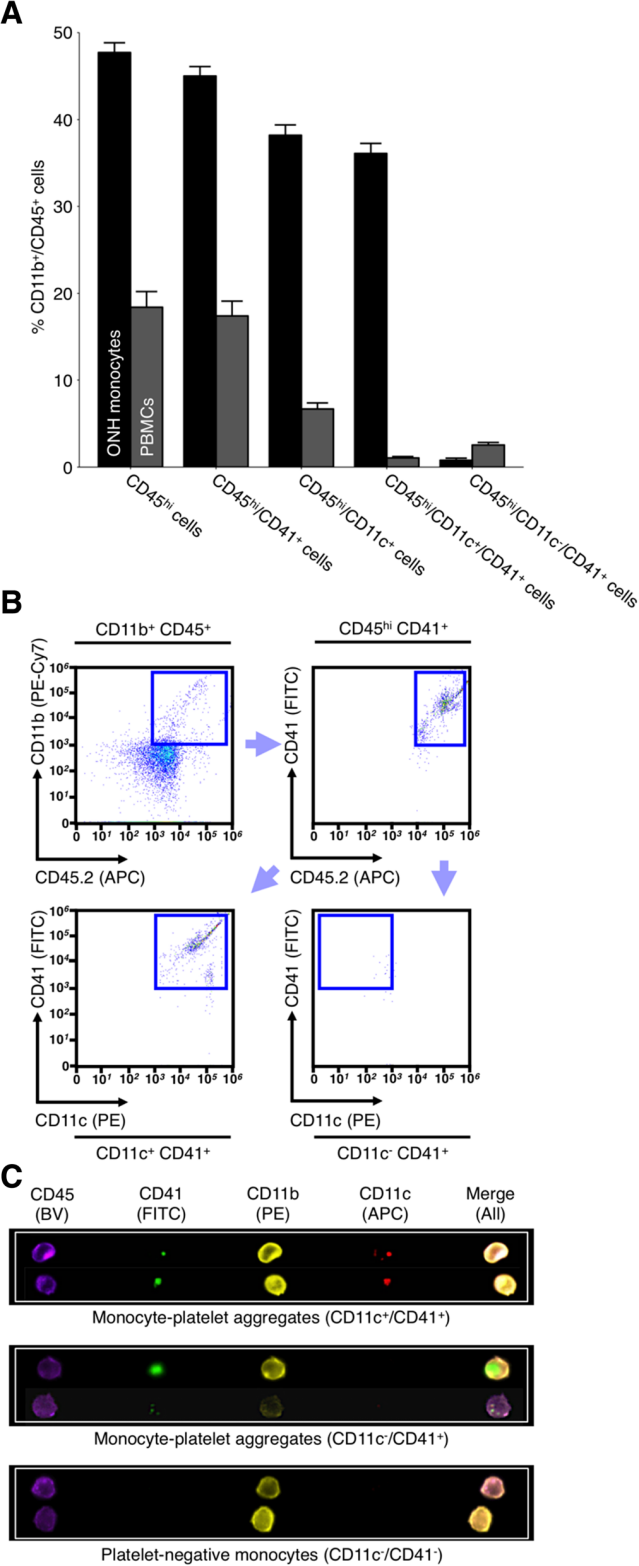
The majority of monocytes entering ONH tissue during glaucomatous progression are platelet bound. We have previously demonstrated that monocytes entering the ONH tissue during glaucoma are CD45^hi^/CD11b^+^/CD11c^+^ (Additional file 1: Figure S1 and [9]). RNA-sequencing analysis of ONH monocytes implicates platelets early in glaucoma pathogenesis. To determine if monocyte-platelet aggregates were present in the ONH in glaucoma flow cytometry was performed. **a** and **b** Antibodies against CD41 (a platelet marker) demonstrated that the majority (> 90%) of CD45^hi^/CD11b^+^/CD11c^+^ ONH monocytes were also platelet bound (*n* = 18 ONHs) compared to < 2% of PBMCs (*n* = 10 peripheral blood samples). **c** When visualized using imaging flow cytometry there were evident platelets bound to monocytes in the ONH (*green*, **c**) (*n* = 4 ONHs). Examples of monocyte-platelet aggregates and platelet negative monocytes are shown. BV = brilliant violet (*blue/violet*), FITC = fluorescein isothiocyanate (*green*), PE = phycoerythrin (*yellow/orange*), APC = allophycocyanin (*red*)

In addition to the role of platelets in endothelial repair, a wealth of evidence demonstrates that platelet binding to monocytes facilitates monocyte entry into tissue by forming monocyte-platelet aggregates that enhance monocyte recruitment to sites of damage [71–74]. In addition, platelets can transfer proteins and RNAs into monocytes affecting their function [75, 76]. Given this evidence we hypothesized that platelet binding to monocytes might play a role in monocyte entry following periods of elevated IOP. To visualize monocyte-like cells and assess platelet binding, we utilized imaging cytometry and immunofluorescence. ONH monocyte samples were stained with an antibody against CD41 (a platelet marker [77]), demonstrating that a large majority (> 90%) of the CD45^hi^/CD11b^+^/CD11c^+^ infiltrating monocyte-like cells in the ONH were platelet-bound (i.e. CD41^+^) (Fig. 3a-c). Immunofluorescence staining on tissues sections through the ONH of D2 and D2-*Gpnmb*^*+*^ control tissue further demonstrated monocyte-platelet aggregates (Additional file 12: Figure S7).

### DS-SILY administration prevents monocyte-like cell entry and provides an extended period of neuroprotection in glaucoma

**Fig. 4:**
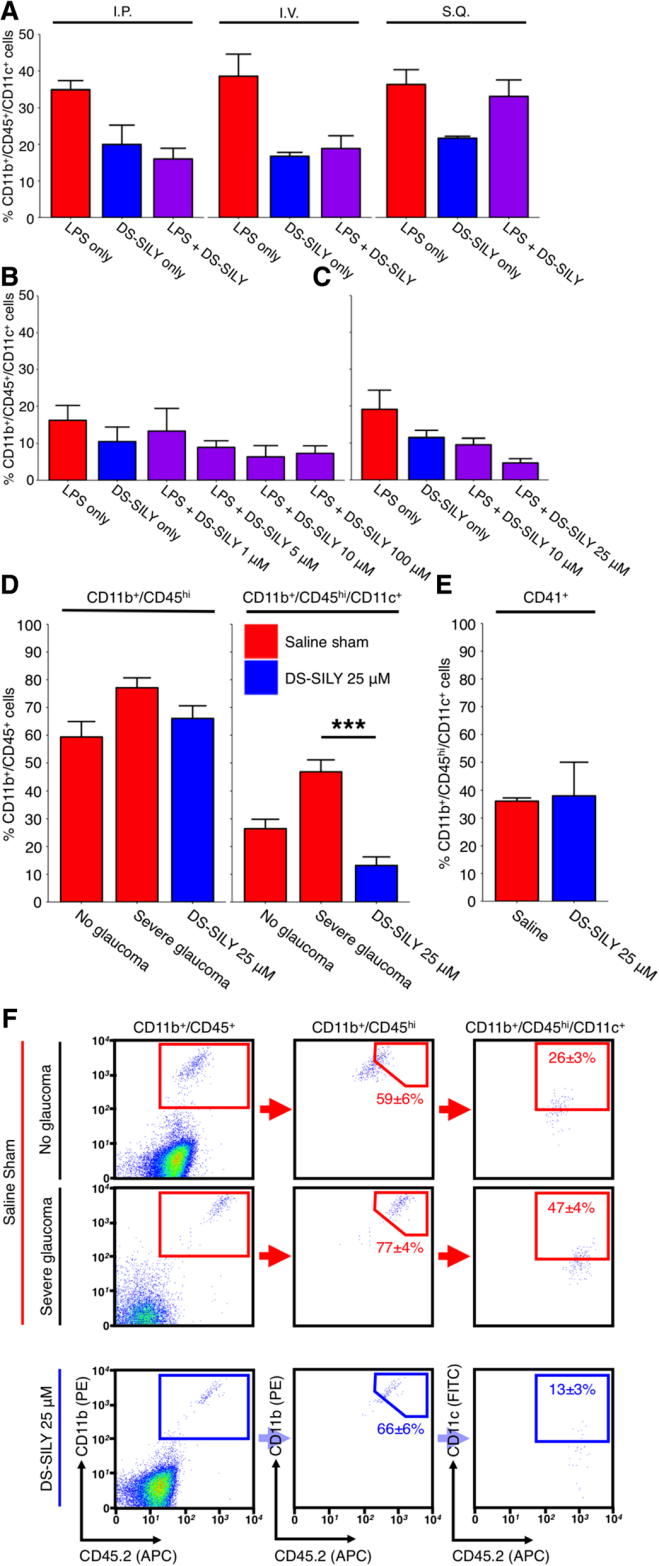
DS-SILY prevents monocyte entry to the ONH. **a**, **b** and **c** 1 μg/g [body weight] LPS delivered by intraperitoneal injection is sufficient to drive monocyte entry into the ONH of young, non-diseased B6 mice after 21 days (**a**, *red* bars). To prevent monocyte entry into the ONH DS-SILY was administered by three different routes; DS-SILY was protective when administered by an intravenous route, (**a**, *purple* bars) in a dose dependent manner (**b**). Although administration by IP had similar potency to IV, its effects were less long-lasting, lasting; IV lasted > 21 days; IP only 3–5 days (not shown) (*n* = 4 for all route testing conditions, *n* > 6 for all concentration testing conditions). DS-SILY administered at 1 μM was used as a sham control (*blue* bars in **a**, **b** and **c**). **c** Further testing in young D2 mice determined that DS-SILY was most potent at 25 μM (*n* = 11 LPS only; 9 DS-SILY only; 23 DS-SILY 10 μM; 8 DS-SILY 25 μM). **d** Subsequently 25 μM DS-SILY was administered every 21 days via intravenous injection to the tail vein of D2 mice starting at 6–7 mo of age. Mice administered DS-SILY and saline sham controls were harvested at 10.5 mo and ONHs assessed for monocyte entry by flow cytometry. DS-SILY robustly protected from CD45^hi^/CD11b^+^/CD11c^+^ monocyte entry (*n* = 24 saline; 23 DS-SILY). **e** A subset of monocytes that still entered in DS-SILY treated ONHs were still platelet positive (*n* = 2 pools of 4 ONHs each). **f** Example flow plots are shown for (**d**). CD11b^+^/CD45^+^ = myeloid-derived cells (i.e. microglia and monocytes in the ONH), CD11b^+^/CD45^hi^ = all monocytes, CD11b^+^/CD45^hi^/CD11c^+^ = infiltrating monocyte-like cells, CD11b^+^/CD45^hi^/CD11c^+^/CD41^+^ = infiltrating monocyte-like cells that are platelet bound (i.e. monocyte-platelet aggregates)

DS-SILY is an engineered peptidoglycan that binds type I collagen. DS-SILY efficiently reduces immune cell and platelet binding by binding to the same targets on endothelial cell surfaces [31, 32]. To evaluate the use of DS-SILY in reducing monocyte-like cell entry into the optic nerve head we tested the effects of DS-SILY administration on CD45^hi^/CD11b^+^/CD11c^+^ infiltrating monocyte-like cells following LPS administration. Administration of 1 μg/g[body weight] LPS to 10 weeks old C57BL/6 J (B6) mice was sufficient to drive the entry of CD45^hi^/CD11b^+^/CD11c^+^ infiltrating monocyte-like cells into the ONH over a 21-day period at a similar number (100–300 cells) to that which occurs in D2 glaucoma [18] (Fig. 4a and b) (without causing optic nerve degeneration as assessed by PPD staining, *data not shown*.). This LPS insult was also able to drive CD45^hi^/CD11b^+^/CD11c^+^ infiltrating monocyte-like cell entry into young, pre-disease D2 eyes (Fig. 4c).

To test if monocyte-like cell entry into the ONH is required for glaucomatous disease progression, we treated mice with the peptidoglycan DS-SILY [31, 32]. This peptidoglycan is well established to inhibit monocyte and platelet binding to collagen at sites of damaged endothelium and can thus lessen vessel wall-platelet and monocyte-platelet interactions, and decrease platelet activation [31, 78]. We used our inducible LPS model of optic nerve inflammation to optimize the route of delivery and dose of DS-SILY using young B6 or D2 mice. Intravenous administration of 25 μM DS-SILY was found to have the most potent anti-inflammatory effect based on inhibition of monocyte entry into the ONH. This effect was potent and long lasting, lasting > 21 days after initial DS-SILY administration (Fig. 4a-c). Using a biotinylated form of DS-SILY (DS-SILY_BIOTIN_ [32]) we confirmed that DS-SILY enters ocular tissues rapidly and was bound to vascular endothelium of the retina, ONH, and choroid (Additional file 13: Figure S8).

To test the long-term effects of DS-SILY treatment, D2 mice were intravenously administered 25 μM DS-SILY in sterile saline (or a saline-only control) once every 21 days from 7 months of age. Tissues were collected at 10.5 and 12 months of age which are key glaucoma time points in this model in our colony, and ONHs were processed for flow cytometry. The number of CD45^hi^/CD11b^+^/CD11c^+^ infiltrating monocyte-like cells was determined in DS-SILY and saline-only control eyes by flow cytometry demonstrating that DS-SILY could potently inhibit monocyte-like cell entry into D2 ONHs (Fig. 4d-f).

Finally, we aimed to determine if inhibition of monocyte-like cell extravasation by DS-SILY administration would be protective against glaucomatous neurodegeneration. Using a sensitive stain for damaged/degenerating axons (PPD) we found that DS-SILY treated optic nerves were robustly protected from damage at 10.5 months of age but this protection was no longer evident at 12 months. Thus, DS-SILY significantly delayed the onset of glaucomatous neurodegeneration.

**Fig. 5:**
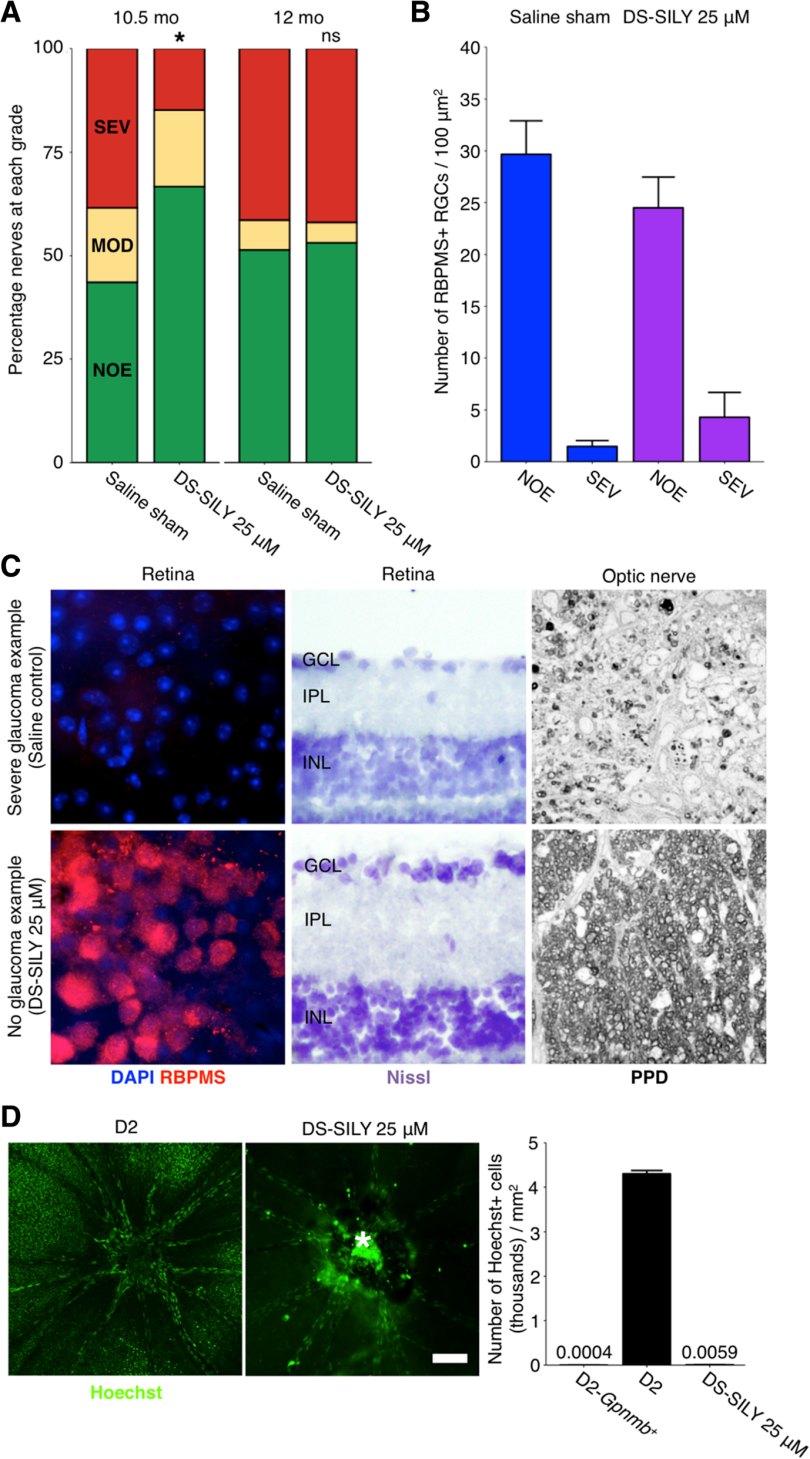
DS-SILY protects from D2 glaucoma at 10.5 mo of age. 25 μM DS-SILY was administered every 21 days via intravenous injection to the tail vein of D2 mice starting at 6–7 mo of age. Mice administered DS-SILY and saline sham controls were harvested at 10.5 and 12 mo and optic nerves and retinas were assessed for glaucomatous neurodegeneration. **a** DS-SILY administration significantly protected D2 eyes from glaucoma at 10.5 mo as assessed by optic nerve assessment (*green* = NOE; no detectable glaucoma but called no or early glaucoma as some eyes have early gene expression changes, *yellow* = MOD; moderate glaucoma, *red* = SEV; severe glaucoma). **b** Agreeing with this, RGC numbers were preserved in protected eyes with no detectable glaucoma (*blue bars* = saline sham, *purple bars* = DS-SILY). Eyes with severe glaucoma had also lost their RGCs indicating that DS-SILY treatment did not uncouple somal and axonal degeneration (SEV bars)). **c** Examples of no glaucoma and severe glaucoma retinas as assessed by RBPMS staining (an RGC marker; *left; n* = 5 for each condition), Nissl staining (*center; n* = 5 for each condition), and optic nerve cross sections assessed by PPD staining (*right; n* = 55 saline 10.5 mo; 64 saline 12 mo; 80 DS-SILY 10.5 mo; 66 DS-SILY 12 mo). *Top row* shows examples of severe retinas from representative eyes with severe optic nerve damage (SEV) as assessed by PPD staining of the optic nerve, *bottom row* shows examples of retinas with no neurodegeneration in the optic nerve (NOE graded nerves). **d** DS-SILY administration potently prevents vascular leakage in the retina at 9–9.5 mo (*n* = 18). Vascular leakage was assessed by an intravenous injection of the nuclear label Hoechst (*green*). Retinas were flat-mounted and Hoechst positive ganglion cell layer nuclei (excluding vascular endothelial cell nuclei) were counted across the retina from 8 representative regions in each eye. The bright region (*) over the optic nerve head in the right hand panel is due to labelling of cells in a residual tuft of hyaloid vasculature over the ONH (as often occurs in mice) and does not indicate increased leakage in the optic nerve. Scale bar = 100 μm. The numbers above D2-*Gpnmb*^*+*^ and DS-SILY 25 μM represent the data points

Our group and others have previously demonstrated that following genetic or pharmacological manipulation of RGCs, axon and soma degeneration can become uncoupled after injury, e.g. soma but not axon survival in *Bax* KO mice [39], and axon but not soma survival in *Wld*^*S*^ rats [79]. To confirm that DS-SILY treatment did not uncouple axon and soma survival, we analysed the soma in mice with no (NOE) or severe axon disease (SEV). As assessed by RBPMS staining, DS-SILY treated eyes with no optic nerve damage (NOE) also had no RGC soma loss while eyes with severe optic nerves had severe loss of soma demonstrating no uncoupling of axon and soma disease following DS-SILY administration (Fig. 5a-c). Importantly, DS-SILY administration did not alter iris disease and ocular hypertension, thus its effects were neuroprotective (Additional file 14: Figure S9). Together, these data suggest that infiltrating monocyte-like cells promote degeneration in DBA/2 J glaucoma. They suggest that platelets promote glaucoma pathogenesis, but further experiments are required to precisely define the role of platelets in this model.

### Elevated IOP induces retinal vasculature permeability

Vascular leakage in the optic disk and retina has been shown to be a component of some human glaucomas, and this may contribute to a neuroinflammatory cascade [80–82]. Given that DS-SILY targets the endothelium and prevents increases in vasculature permeability in other model systems, we explored whether vasculature permeability changes occur in D2 mice and whether DS-SILY’s actions may, in part, be due to its protective effects on the endothelium.

The ONH is supplied by backwards capillaries emanating from the choroid, directly beneath the retinal pigment epithelium, RPE, and surrounding the ONH [83]. These vessels are highly fenestrated and thus ‘leaky’ [84–86], and may confound any experiments assessing vascular leakage in the ONH. In contrast to the ONH, the neural retina is protected from the central blood supply by the blood-retinal barrier (BRB) that closely resembles the blood brain barrier, containing tight junctions that limit the passage of micro- and macro- molecules. The BRB consists of two barrier sites: the RPE (the outer barrier) and the retinal capillary endothelial cells (the inner barrier) [83]. We have previously demonstrated that monocytes infiltrate into the retina as well as the ONH during D2 glaucoma [18]. Therefore, as the vasculature of the choroid is inherently leaky, we assessed the retinal vasculature for changes in permeability (i.e. assessing the inner BRB). Utilizing D2, D2-*Gpnmb*^+^, and radiation-treated D2 mice we used the DNA binding dye Hoechst 33342 (small molecular weight of 453 g/mol) as an indicator of vascular permeability. We chose a small molecular weight dye to assess vascular permeability versus gross vascular leakage [86]. We discovered that retinas of D2 mice at 9 months of age (but not D2-*Gpnmb*^+^) are permeable to intravenously injected Hoechst (Additional file 16: Figure S10). Radiation therapy did not prevent endothelial permeability to Hoechst suggesting that the protection mediated by radiation therapy is downstream to, or independent of, vascular permeability. Further work and tool development outside of the scope of this study are required to fully assess vasculature permeability in the ONH during glaucomatous insults.

Vascular leakage was present in the retinas of 9 month old D2 mice (prior to RGC loss), but not age-matched D2-*Gpnmb*^*+*^ mice, suggesting that vascular permeability may be driven by changes in IOP and possibly by monocyte-platelet aggregates. Although platelets are conventionally known to promote vascular integrity, they are capable of inducing and maintaining permeability [87, 88]. Vascular permeability in inflamed joints is abrogated when platelets are absent [89]. Thus, we assessed the effects of DS-SILY on retinal vascular permeability in D2 glaucoma. D2 mice were administered DS-SILY (as above) from 7 months of age and were intravenously injected Hoechst at 9 months of age. Analysis of DS-SILY administered retinas showed no leakage, similar to D2-*Gpnmb*^+^ controls, demonstrating that DS-SILY was able to potently inhibit the development of IOP-induced retinal vascular permeability (Fig. 5d).

### *Itgam* (CD11b) mediates monocyte-like cell entry and optic nerve damage in glaucoma

We next sought to understand the upstream molecules that may affect monocyte identity and activity. Enrichment analysis of DE gene Modules 1–5 using DiRE (distant regulatory elements of co-regulated genes [90]) allowed further insight into upstream effectors/transcription factors (Additional file 15: Table S6). Of primary interest, Module 2 favoured monocyte development and differentiation (e.g. *Gata3*, *Vdr*). The activated vitamin D receptor (VDR) is a master regulator for monocytic differentiation, inducing differentiation of monocytes into resident macrophage populations [91, 92]. Vitamin D_3_ has been shown to increase cell surface expression of *Itgam* (integrin subunit alpha m or CD11b), the α subunit of CR3 (complement receptor 3) [92, 93]. We have previously reported important roles for the complement pathways in glaucoma [5–7, 9, 94] and the genetic subunits of CR3 (*Itgam* and *Itgb2*) are both highly expressed in our infiltrating monocytes, represented in the top 1.5% of expressed genes.

*Itgam* encodes a cell adhesion molecule that is central in monocyte entry into tissues including adhesion of monocytes to fibrinogen and to platelets [95–98]. In addition, CD11b is upregulated on human monocytes following platelet adhesion [74]. Given these data, including the fact that *Itgam* is one of the highest expressed genes in our monocyte populations, *Itgam* is an excellent candidate to modulate IOP-induced monocyte entry. To test the role of *Itgam*, we generated D2 mice carrying a null allele of *Itgam* (D2.*Itgam*^−/−^). D2.*Itgam*^−/−^ mice had no detectable levels of CD11b protein, and had iris disease that was indistinguishable from wild-type controls and ocular hypertension that was indistinguishable from wild-type controls at 8 and 10 months of age but tended to persist longer (*P* < 0.01 at 12 months of age) (Additional file 17: Figure S11).

**Fig. 6:**
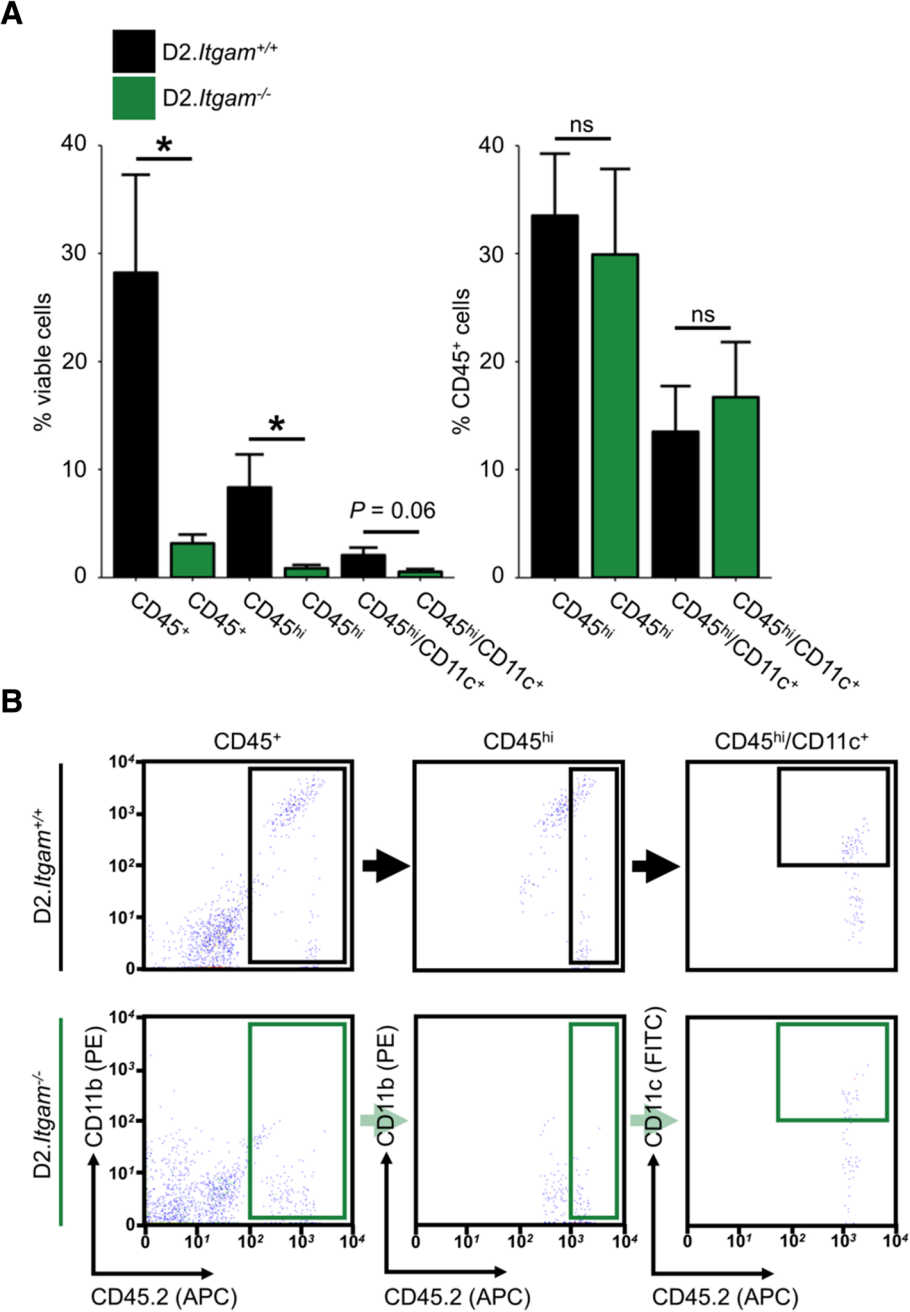
Genetic ablation of CD11b (*Itgam*) decreases monocyte number in the ONH. To determine whether genetic ablation of *Itgam* affects monocyte entry into the ONH during glaucoma, D2.*Itgam*^*−/−*^ mice and genetic controls were harvested at 10.5 mo and ONHs assessed for monocytes by flow cytometry. **a** CD45^+^ and CD45^hi^ cell number in the ONH was significantly decreased in *Itgam*^*−/−*^ mice (**a**, *left*). Homozygous knockout mice lack CD11b and so the gating strategy compared to % viable cells. The relative proportions of CD45^hi^ and CD45^hi^/CD11c^+^ types of myeloid derived cells were not changed (**a**, *right*) (*n* = 12 (D2.*Itgam*^*+/+*^), 10 (D2.*Itgam*^*−/−*^)). **b** Example flow plots and gating strategy

We next assessed monocyte numbers in the optic nerve head by flow cytometry in D2.*Itgam*^−/−^ and wild-type control mice. CD11b was not used for gating in this analysis, as D2.*Itgam*^−/−^ mice had no detectable levels of CD11b. Thus, CD45^hi^ cell numbers were regarded as representative of monocytes for this experiment. D2.*Itgam*^−/−^ had significantly diminished numbers of all myeloid derived cells in the ONH (i.e. CD45^+^ and CD45^hi^ cells, Fig. 6a-b), supporting a diminished number of CD45^hi^/CD11c^+^ cells in the ONH. Very few CD45^hi^/CD11c^+^ cells were observed in the ONH of D2.*Itgam*^−/−^ mice (0.5% of viable cells, Fig. 6a), but this did not reach statistical significance (*P* = 0.06). Collectively, these data suggest that CD11b is an important mediator of monocyte-like cell entry in D2 glaucoma.

**Fig. 7:**
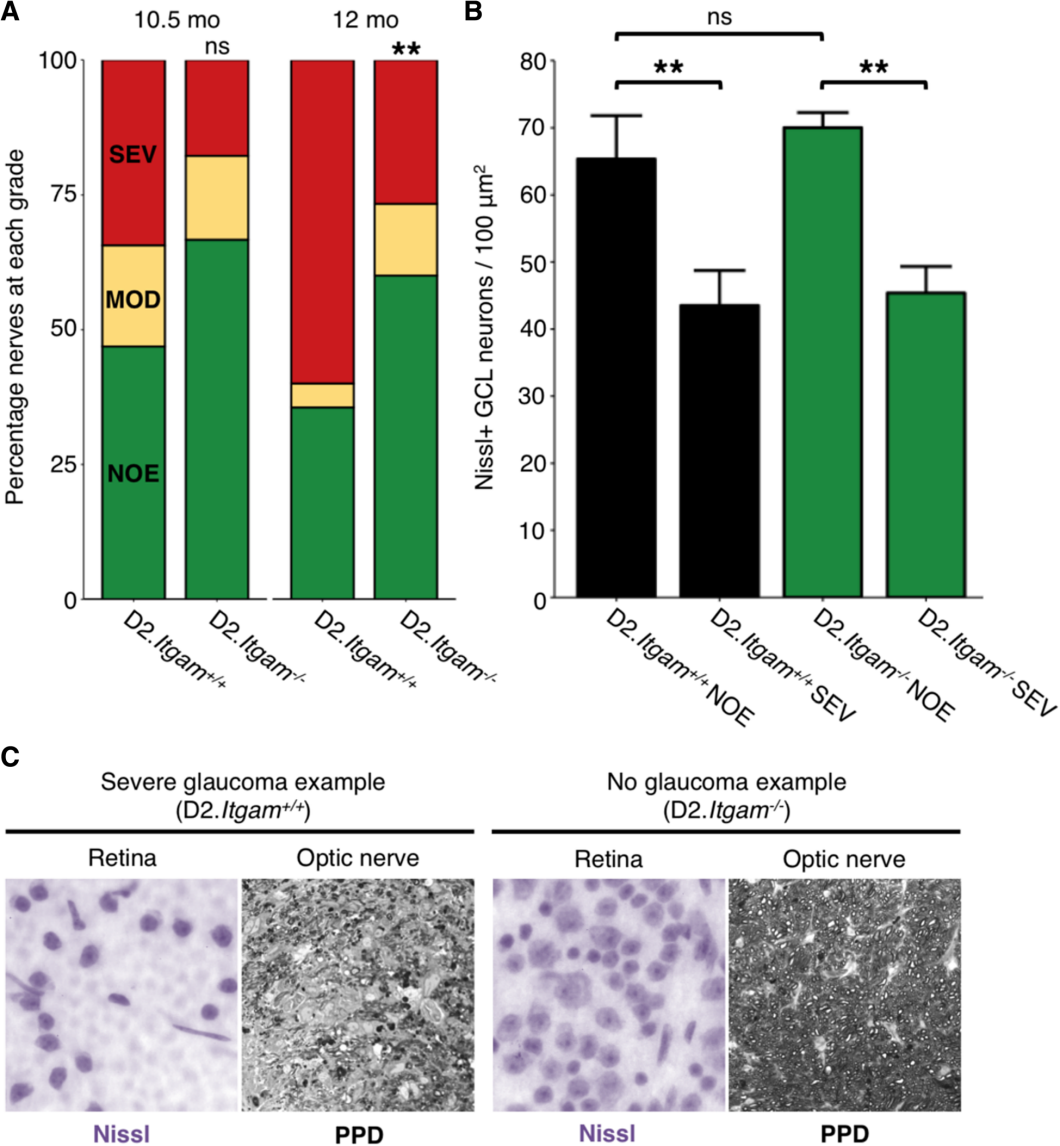
Genetic ablation of CD11b (*Itgam*) protects from glaucoma to aged time points. **a** D2.*Itgam*^*−/−*^ mice are significantly protected from glaucoma as determined by optic nerve assessment (*green* = NOE; no detectable glaucoma but called no or early glaucoma as some of these eyes have early gene expression changes, *yellow* = MOD; moderate glaucoma, *red* = SEV; severe glaucoma). **b** Agreeing with this, RGC numbers were preserved in eyes with no detectable glaucoma. Eyes with severe glaucoma had also lost their RGCs indicating that loss of CD11b did not uncouple somal and axonal degeneration. **c** Example optic nerves and retinas. *Left* shows examples of severe retinas from eyes with severe optic nerve damage (SEV) as assessed by PPD staining of the optic nerve, *right* shows examples of retinas with no neurodegeneration in the optic nerve (NOE graded nerves). (Soma counts, *n* = 6 for each condition; optic nerve analysis as assessed by PPD staining (*right*) *n* = 32 D2.*Itgam*^*+/+*^ 10.5 mo; 45 D2.*Itgam*^*+/+*^ 12 mo; 45 D2.*Itgam*^*−/−*^ 10.5 mo; 45 D2.*Itgam*^*−/−*^ 12 mo)

Genetic ablation of *Itgam* robustly prevented monocyte-like cell entry, and so we next assessed optic nerves and retinas for glaucomatous neurodegeneration. D2-*Itgam*^−/−^ eyes had a greatly decreased frequency of monocyte infiltration (Fig. 6a) that corresponded with a significantly decreased risk of developing severe glaucoma (Fig. 7a-c). This protection against glaucoma was significant out to later disease time points (12 months) as assessed by PPD staining of the optic nerve. Axonal and somal disease process were not uncoupled by genetic removal of *Itgam,* which protected both RGC soma and axons (Fig. 7b-c). Collectively, these data suggest that monocyte-like cell entry is a key mediator of optic nerve degeneration in DBA/2 J glaucoma.

## Discussion

Our group and others have identified the ONH as a critical site of insult and damage early in glaucoma [4, 19, 99–105]. The ONH contains a network of glial cells that support RGC axons and a neurovascular network acting as a blood-retinal barrier. DBA/2 J glaucoma appears to be (at least partially) a neuroinflammatory disease with damage coordinated through dysfunctional RGCs, reactive glial cells, and the infiltration of monocyte-like immune cells from the blood [3, 18, 24, 94]. Recent work has demonstrated that CD163^+^ macrophage numbers increase in the ONH of human patients with both mild and severe glaucomatous optic neuropathy, and thus, immune-cell infiltration or expansion may be an important part of at least some human glaucomas [4]. However, the roles and molecular profiles of infiltrating monocyte-like cells are not adequately demonstrated by previous publications, and thus are the major focus of the current study.

We first confirmed monocyte-like cell extravasation by cell labelling using CFDA within the spleen (with labelled cells homing to the ONH) and by flow cytometry for cells with high expression of CD45 (CD45^hi^) in the ONH [18]. CD45 is a well-established marker for circulating cells derived from bone marrow, with infiltrating monocytes being CD45^hi^ and resident microglia being CD45^lo^ [106, 107]. We found that monocyte numbers are increased in the ONH in ocular hypertensive DBA/2 J mice compared to normotensive D2-*Gpnmb*^*+*^ mice (Additional file 1: Figure S1) [5, 18]. Although it is possible that *Gpnmb* genotype modulates immune cell entry, these data suggest ocular hypertension causes neuroinflammation in the ONH in DBA/2 J mice. An increase in neuroinflammatory molecules in the ONH is seen in many animal models of induced ocular hypertension. However, the effect of ocular hypertension on monocyte entry, in DBA/2 J mice and in other glaucoma models, has yet to be fully determined. Infiltrating monocyte-like cells are predicted to produce damaging molecules that drive neurodegeneration in DBA/2 J glaucoma, including endothelin 2 and complement component C1. Altering endothelin 2 or complement C1 is protective in this model supporting a role for these monocyte-like cells in this disease [6, 7, 9]. In fact, adding endothelin 2 to radiation treated DBA/2 J mice (mice that are typically protected from glaucoma) induces glaucoma-like neurodegeneration [7, 9, 18].

In the current datasets, optic nerve head monocyte-like cells highly express various complement genes (including *C1qa*, *C1qb*, *C1qc*, *C3*, and *C3ar1*), and complement activation is implicated in human glaucoma [108–110]. Inhibiting C1 either through genetic ablation (*C1qa*) or pharmacologically (C1 esterase inhibitor) prevents RGC dendritic and synaptic changes during glaucoma in both DBA/2 J mice and a rat bead model of ocular hypertension [6, 9]. This highlights the importance of these factors as a more general response to periods of elevated IOP rather than a DBA/2 J specific process [110]. Our group have also previously demonstrated that complement component C3 is protective while C5 is damaging in the DBA/2 J model [5, 94]. More work is required to elucidate the full role of complement pathway molecules in specific cell types during glaucoma and the current data open an avenue for functionally exploring additional key molecules such as C3ar1.

To further assess the role of infiltrating monocyte-like cells in DBA/2 J glaucoma, and as platelet interaction signatures were detected by our gene expression studies, we decided on a strategy using DS-SILY. DS-SILY is composed of dermatan sulfate, the polysaccharide chain from decorin, with covalently attached collagen binding peptides derived from the non-integrin platelet receptor for type I collagen [78, 111, 112]. Due to the absence of the core protein sequence and structure of decorin, DS-SILY does not mimic other functions of decorin, such as TGF-β1 regulation [113] and thus DS-SILY’s neuroprotective effects are unlikely to be due to effects directly on RGCs or astrocytes. DS-SILY has been shown to inhibit platelet and immune cell binding to collagen in damaged vascular endothelium in a pig model of angioplasty and in human vascular smooth muscle cells [31, 32, 78, 114]. Importantly, we show that DS-SILY prevented the accumulation of CD45^hi^/CD11b^+^/CD11c^+^ monocyte-like cells in the ONH and retina of DBA/2 J mice and is neuroprotective.

In the DS-SILY experiment, the, saline treated sham controls appear to have a lower incidence of severe optic nerve degeneration than is typical for DBA/2 J mice at the 12 month time point. It is known that the distribution of glaucoma incidence and severity varies over time and between colonies of DBA/2 J mice, as is true for a variety of other complex diseases. The reason for this is not well understood and likely involves uncontrollable environmental differences that vary over time. For this reason, it is important to limit comparisons between groups that were bred, aged, and analysed at the same time as each other, i.e. within an experiment. Importantly, there was no significant difference between the 12 month control nerves in the D2.*Itgam* and DS-SILY experiments (*Fisher’s exact* test, *P* = 0.15).

DS-SILY administration also potently inhibited retinal vascular permeability in DBA/2 J mice as assessed by Hoechst dye leakage in the retina. Vascular dysfunction and leakage from the endothelium are associated with ocular hypertension and have both been recorded in human glaucoma [80–82]. Although further experiments are required to fully understand how DS-SILY protects from glaucoma, these studies implicate endothelial dysfunction in the entry of CD45^hi^/CD11b^+^/CD11c^+^ monocyte-like cells during glaucoma pathogenesis. Given that neuroinflammation and CD45^hi^/CD11b^+^/CD11c^+^ monocyte–like cells are being implicated in other diseases, our data suggest that DS-SILY could offer protection in other neuroinflammatory conditions.

*Itgam* is one of the most highly expressed genes in the monocyte-like cells that enter the optic nerve during DBA/2 J glaucoma. *Itgam* encodes CD11b, a key molecule for extravasation and that binds to fibrinogen [95]. Removing CD11b would be predicted to limit monocyte entry and immune cell binding. Supporting a damaging role for monocyte-like cells in DBA/2 J glaucoma, we show that genetic ablation of *Itgam* decreased their entry and prevented optic nerve degeneration through to late time points. However, monocyte-like cells are not the only cells that express CD11b in the ONH. DBA/2 J ONHs and retinas also contain reactive / activated microglia (CD45^lo^/CD11b^+^ cells) [3, 7–9, 61]. It is possible, that as an alternative mechanism to preventing monocyte entry, removing CD11b limits the damaging actions of microglia. This offers a future avenue of research using specific *cre*-lines to explore the actions of CD11b in a cell-type specific setting. Nevertheless, pharmacological strategies to inhibit CD11b (for example antibody therapies or local gene therapy) would make for potentially exciting human therapeutic strategies in neuroinflammatory disease. CD11b is not the only integrin expressed by infiltrating monocyte-like cells, and it is not surprising that removal of CD11b alone did not fully prevent the glaucoma. Future experiments could investigate inhibition of both CD11b and CD11c *(Itgam-Itgax* double knockout) to further target the infiltrating monocyte-like cells in DBA/2 J glaucoma and provide further detail about the role of the monocyte-like cells.

With increasing age and exposure to elevated IOP, both D2.*Itgam*^*−/−*^ mice and DS-SILY-treated mice developed severe forms of glaucoma and it is not surprising that these individual treatments were less effective than radiation therapy [23]. It may be that radiation therapy protects via additional mechanisms to monocyte entry alone. In the DS-SILY experiments, the dose was optimized based on an acute inflammatory event and thus may also need to be further calibrated in a chronic inflammatory setting. In addition, retinal ganglion cell soma counts for these experiments were based on RBPMS staining, and it is possible that RBPMS down-regulation may have disguised some additional protective effect. Nevertheless, DS-SILY treatment robustly prevented optic nerve degeneration at early disease time points. It is unlikely that a sole factor (e.g. monocyte inhibition) will be sufficient for complete neuroprotection in glaucoma. We have previously demonstrated that stopping RGC specific neurodegenerative events is robustly protective even when the neuroinflammatory insult continues (e.g. *Wld*^*S*^ [40], *Nmnat1* gene therapy [33]). Thus, there are likely RGC specific insults and RGC intrinsic events that continue to occur despite monocyte depletion in DS-SILY treated mice and D2.*Itgam*^−/−^ mice. Further neuroprotective strategies could include treatments that target both intrinsic and extrinsic factors to RGC survival. It is likely that immunomodulation will need to be used in combination with other neuroprotective strategies for optimal therapeutic effect.

Studies in various neurodegenerative diseases, particularly Alzheimer’s disease, suggest that myeloid cells are playing an important role in disease onset and / or progression [115–117]. One study, using single cell profiling, suggests CD11b^+^/CD11c^+^ myeloid cells may be critical for transition from a homeostatic to disease-promoting state [116]. Since we show that similar cells are present in DBA/2 J glaucoma, further studies to understand the transcriptional / activation states of CD11b^+^/CD11c^+^ monocyte-like cells in glaucoma are warranted. Although it is not yet clear if modulating myeloid cell activation or depleting / blocking entry of these cells is the most appropriate strategy for neuroprotection in glaucoma such studies may lead to important new therapeutics. Further work testing and confirming myeloid cell activity in additional models of glaucoma is needed.

## Conclusions

Considering these findings, IOP-induced neuroinflammation and entry of damaging monocyte-platelet aggregates into the ONH appear to be important contributors to early glaucomatous damage. Targeting monocyte infiltration, platelet aggregation and activation, and / or vascular dysfunction may offer novel therapeutic interventions in glaucoma and other neuroinflammatory conditions.

## Additional files

Additional file 1: **Figure S1.** CD45^hi^/CD11b^+^/CD11c^+^ monocyte-like cells infiltrate early in D2 glaucoma. At ~ 9 mo the majority of D2 eyes in our colony have experienced periods of high IOP. We have previously demonstrated that at this 9 mo time point there are early metabolic and transcriptomic differences in retinal ganglion cells that occurs prior to detectable axon degeneration [33]. Analysing ONH from D2 mice at this time point confirms that monocytes (CD45^+^/CD11b^+^/CD11c^+^) enter the ONH tissue during this early period of metabolic decline. These cells do not increase in either genetic control D2-*Gpnmb*^*+*^ or irradiated treat D2 ONHs (mice that are protected from neurodegeneration but still have a normal D2 front-of-the-eye disease [18]) suggesting that these infiltrating cells initiate a damaging cascade during glaucoma progression. *n* = 25 (D2-*Gpnmb*^*+*^), 45 (D2), 25 (irradiated D2). (TIFF 1162 kb)

Additional file 2: **Figure S2.** FAC sorting of CD45^hi^/CD11b^+^/CD11c^+^ monocyte-like cells that enter the ONH. For FAC sorting of monocyte populations ONH samples were stained with an antibody cocktail (see Materials and methods) to eliminate other cell types. Forwards and sidewards scattering identify relevant events (**A** and **B**) that are gated to identify live cells (**C** and **D**). Monocytes were identified as gated CD45hi/CD11b/CD11c + (and CD34^−^/GFAP^−^) events (**E-I**). Monocytes were distinguished from resident microglia by CD45 (monocytes being CD45^hi^ and microglia being CD45^lo^ [107], **F** and **G**). Despite the low cell input (< 100 cells per sample, see Additional file 1: Figure S1), high quality, non-degraded, non-contaminated mRNA was successfully collected (**J**). (TIF 4183 kb)

Additional file 3: **Figure S3.** Enriched genes in monocyte populations. Highest enriched genes for ONH Monocytes Group 1 (*left*) and PBMCs (*right*) are shown. *Red* = highest expression, *blue* = lowest expression. Analysis was performed using Gene Set Enrichment Analysis (GSEA) [118]. (TIFF 10715 kb)

Additional file 4: **Figure S4.** Inflammatory monocyte markers at mRNA level. Common mouse monocyte markers at an mRNA level in ONH Monocytes vs. PBMCs by fold change from control (**A**) and by normalized CPM (counts per million) (**B**). DE genes (FDR < 0.05) are shown in *red*. (*Itgam* encodes CD11b, *Itgax* encodes CD11c, *Ptprc* encodes CD45). (TIFF 3547 kb)

Additional file 5: **Table S1.** Pathway analysis of DE genes in ONH monocytes. (CSV 6 kb)

Additional file 6: **Figure S5.** KEGG analysis of enriched gene sets in ONH Monocytes Group 1. Scatter plots of genes by fold change from PBMCs (*y*) and CPM (*x*). (**A**) Genes enriched in KEGG: mmu04662, B cell receptor signalling, (**B**) KEGG: mmu04660, T cell receptor signalling, and (**C**) KEGG: mmu04514, cell adhesion molecules. *Grey* = not DE, *red* = DE (FDR < 0.05). DE genes at FDR < 0.01 with a log_2_ CPM > 5 are named. (TIFF 6547 kb)

Additional file 7: **Table S2.** Pathway analysis of differentially spliced transcripts (MATS analysis of ONH Monocytes Group 1 vs. PBMCs). (CSV 6 kb)

Additional file 8: **Table S3.** Alternately expressed transcripts (MATS analysis of ONH Monocytes Group 1 vs. PBMCs). (CSV 14 kb)

Additional file 9: **Figure S6.** Modular analysis of DE genes. Modules 1–5 were generated by hierarchical clustering (*Spearman’s*) based on DE genes (FDR < 0.05) between ONH Monocytes Group 1 vs. PBMCs. Each module is color-coded and the number of genes in the module is shown. Individual modules then underwent further analysis (see Results). (TIF 10259 kb)

Additional file 10: **Table S4.** Pathway analysis of DE gene Modules 1-5 (ONH Monocytes Group 1 vs. PBMCs). (CSV 7 kb)

Additional file 11: **Table S5.** KEGG pathway and PANTHER classification analysis of DE gene Modules 1-5 (ONH Monocytes Group 1 vs. PBMCs). (CSV 3 kb)

Additional file 12: **Figure S7.** Monocytes entering the ONH during glaucoma are platelet bound. ONHs assessed by flow cytometry demonstrate that the majority of monocytes entering the ONH during glaucoma pathogenesis are platelet bound (Fig. 3). To further confirm that monocyte-platelet aggregates were present in the ONH in glaucoma, immunofluorescence using antibodies against CD41 (a platelet marker, *green*) and CD11b (a monocyte maker, *red*) was performed in longitudinal sections of D2 and D2-*Gpnmb*^*+*^ ONH tissue (*n* = 4 / group, see upper right panel for ONH location for all upper and middle panels). The majority of monocytes that enter the ONH during glaucoma in D2 eyes are platelet-bound. APC = allophycocyanin (*red*), FITC = fluorescein isothiocyanate (*green*)*.* Scale bar = 100 μm. (TIF 9562 kb)

Additional file 13: **Figure S8.** DS-SILY binds to collagen in the retina, ONH, and surrounding vasculature. Eyes from mice that had been administered DS-SILY_BIOTIN_ were assessed by immunofluorescence. DS-SILY_BIOTIN_ clearly makes it to the eye and binds to inner retina vasculature (GCL, IPL), optic nerve head vasculature, and the collagen of rod outer segments (*red*). No fluorescence is seen in control retina and ONH (**B**) (*n* = 5 for both conditions). GCL (ganglion cell layer), IPL (inner plexiform layer), INL (inner nuclear layer), OPL (outer plexiform layer), ONL (outer nuclear layer). (TIFF 3770 kb)

Additional file 14: **Figure S9.** Mice administered DS-SILY have IOP elevating anterior segment disease similar to saline sham controls. IOP profiles (**A**) and clinical presentation of iris disease (**B**) (*n* > 40 all conditions). IOP is not significantly different between cohorts within the same age-group. Iris disease (iris pigment dispersion resulting in asynchronous ocular hypertension) progressed at a similar rate and reached a severe state in all groups within the same time-frame. For boxplots, the upper and lower hinges represent the upper and lower quartiles. The centerline of each diamond (*red*) represents the mean, and the upper and lower diamond points represent 95% confidence intervals of the mean. (TIFF 7256 kb)

Additional file 15: **Table S6.** Enrichment analysis of DE gene Modules 1–5 using DiRE. (CSV 4 kb)

Additional file 16: **Figure S10.** Vascular leakage occurs early in D2 glaucoma. To explore retinal vascular damage and leakage in glaucoma, mice (9–9.5 mo of age) were intravenously injected with the DNA binding dye Hoechst. Retinas were then flat-mounted and Hoechst+ nuclei (excluding vascular endothelial cell nuclei identified by their more elliptical shape) were counted from 8 representative images of the retina. In control D2.*Gpnmb*^*+*^ mice Hoechst was bound only to cells within the vasculature (*green*, i.e. no leakage into surrounding retina). However, by 9 mo of age leakage was evident in the retinas of D2 mice (as demonstrated by nuclear labelling of all cells surrounding the retinal vasculature). Vascular leakage was evident in retinas from irradiated D2 mice that are protected from monocyte entry and glaucomatous neurodegeneration suggesting that monocyte entry in glaucoma (at least in part) must be driven by an active inflammatory process. (*n* = 30 D2-*Gpnmb*^*+*^; 18 D2; 20 irradiated D2). Scale bar = 100 μm. The number (0.0004) above D2-*Gpnmb*^*+*^ represents the data point. (TIF 4988 kb)

Additional file 17: **Figure S11.** D2.*Itgam*^*−/−*^ mice have IOP elevating anterior segment disease similar to wild-type controls. IOP profiles (**A**) and clinical presentation of iris disease (**B**) (*n* > 40 all conditions). IOP is not significantly different between cohorts within the same age-group at 8 and 10 months of age, but D2.*Itgam*^*−/−*^ eyes were more resistant to the IOP decline that usually occurs around 12 months of age (*P* < 0.01). Iris disease (iris pigment dispersion resulting in asynchronous ocular hypertension) progressed at a similar rate and reached a severe state in all groups within the same time-frame. For boxplots, the upper and lower hinges represent the upper and lower quartiles. The centerline of each diamond (*red*) represents the mean, and the upper and lower diamond points represent 95% confidence intervals of the mean. (TIFF 5109 kb)

## Abbreviations

ACK: Ammonium-chloride-potassium buffer
BRB: Blood-retinal barrier
DE: Differentially expressed
EDTA: Ethylenediaminetetraacetic acid
FACS: Fluorescence-activated cell sorting
FDR: False discovery rate
HBSS: Hanks’ balanced salt solution
HC: Hierarchical clustering
IOP: Intraocular pressure
LPS: Lipopolysaccharide
MOD: Moderate
NOE: No or early
OCT: Optimal cutting temperature
ON: Overnight
ONH: Optic nerve head
PBMC: Peripheral blood monocyte
PBS: Phosphate buffered saline
PPD: Paraphenylenediamide
RGC: Retinal ganglion cell
SEV: Severe
TMM: Trimmed mean of M values
