# Supplementary figures and images for: Inhibition of monocyte-like cell extravasation protects from neurodegeneration in DBA/2J glaucoma
Source: Mol Neurodegener. 2019 Jan 22;14:6. doi: 10.1186/s13024-018-0303-3 (PMC6341618; doi:10.1186/s13024-018-0303-3)

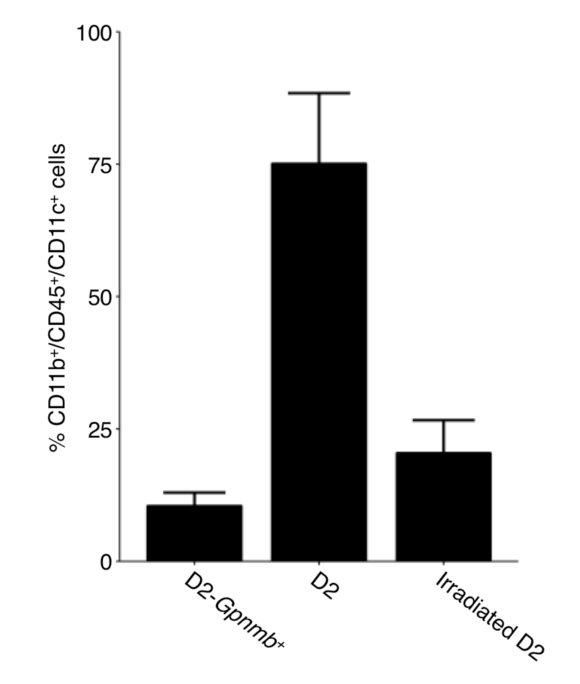

Supplement: Supplementary file 1 — Figure S1. CD45hi/CD11b+/CD11c+ monocyte-like cells infiltrate early in D2 glaucoma. At ~ 9 mo the majority of D2 eyes in our colony have experienced periods of high IOP. We have previously demonstrated that at this 9 mo time point there are early metabolic and transcriptomic differences in retinal ganglion cells that occurs prior to detectable axon degeneration [33]. Analysing ONH from D2 mice at this time point confirms that monocytes (CD45+/CD11b+/CD11c+) enter the ONH tissue during this early period of metabolic decline. These cells do not increase in either genetic control D2-Gpnmb+ or irradiated treat D2 ONHs (mice that are protected from neurodegeneration but still have a normal D2 front-of-the-eye disease [18]) suggesting that these infiltrating cells initiate a damaging cascade during glaucoma progression. n = 25 (D2-Gpnmb+), 45 (D2), 25 (irradiated D2). (TIFF 1162 kb) [file 13024_2018_303_MOESM1_ESM.tiff]

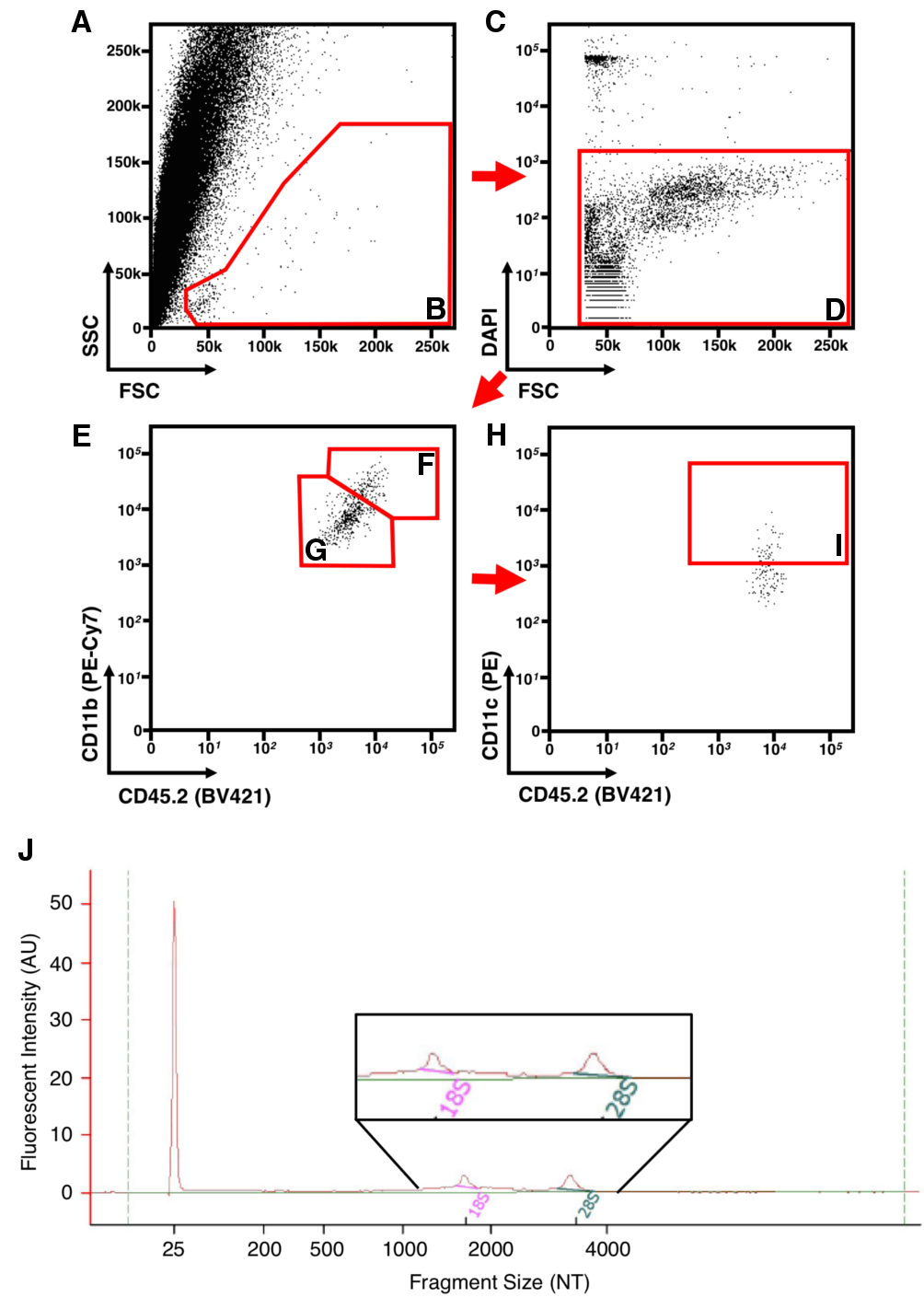

Supplement: Supplementary file 2 — Figure S2. FAC sorting of CD45hi/CD11b+/CD11c+ monocyte-like cells that enter the ONH. For FAC sorting of monocyte populations ONH samples were stained with an antibody cocktail (see Materials and methods) to eliminate other cell types. Forwards and sidewards scattering identify relevant events (A and B) that are gated to identify live cells (C and D). Monocytes were identified as gated CD45hi/CD11b/CD11c + (and CD34−/GFAP−) events (E-I). Monocytes were distinguished from resident microglia by CD45 (monocytes being CD45hi and microglia being CD45lo [107], F and G). Despite the low cell input (< 100 cells per sample, see Additional file 1: Figure S1), high quality, non-degraded, non-contaminated mRNA was successfully collected (J). (TIF 4183 kb) [file 13024_2018_303_MOESM2_ESM.tif]

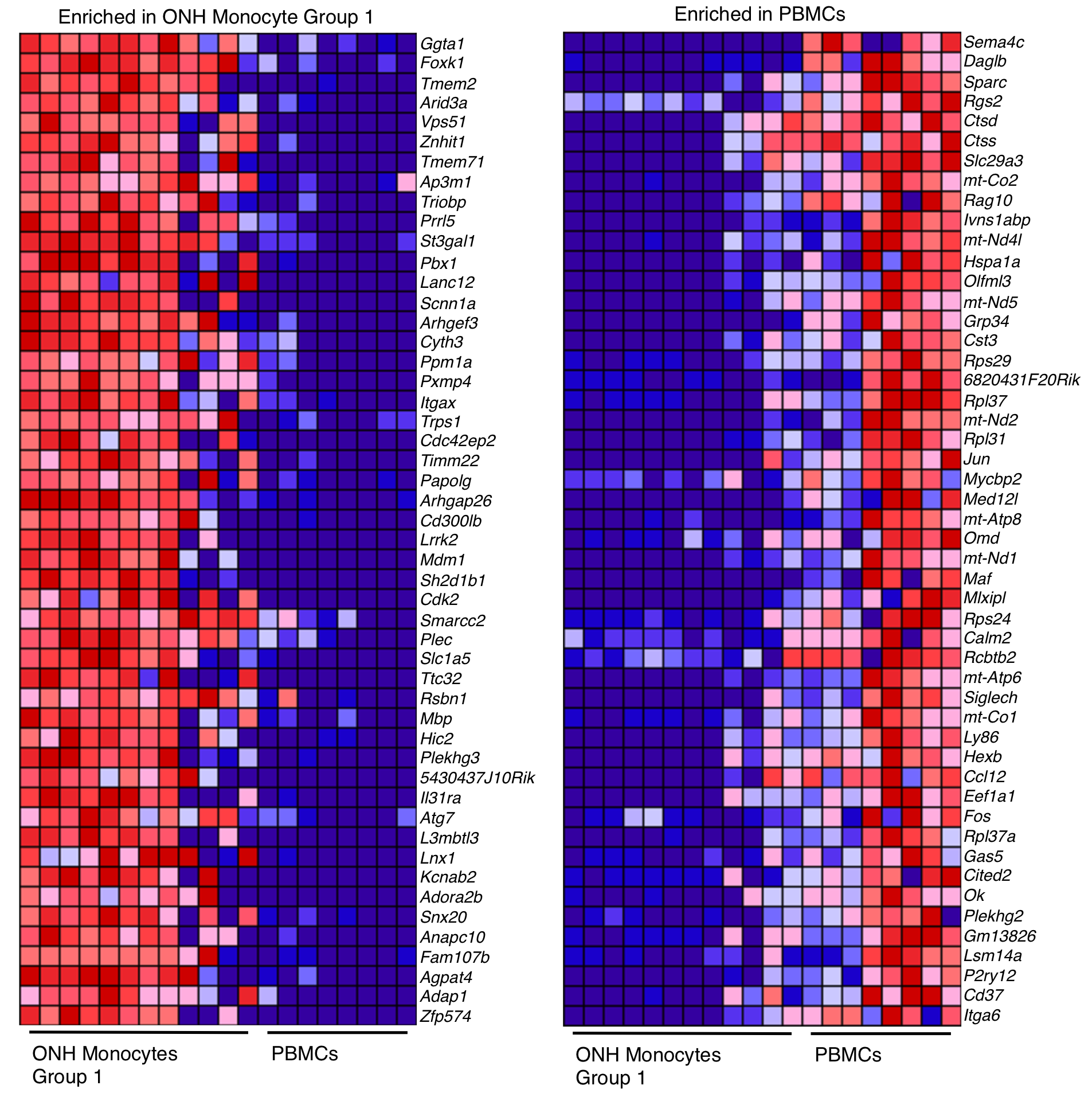

Supplement: Supplementary file 3 — Figure S3. Enriched genes in monocyte populations. Highest enriched genes for ONH Monocytes Group 1 (left) and PBMCs (right) are shown. Red = highest expression, blue = lowest expression. Analysis was performed using Gene Set Enrichment Analysis (GSEA) [118]. (TIFF 10715 kb) [file 13024_2018_303_MOESM3_ESM.tiff]

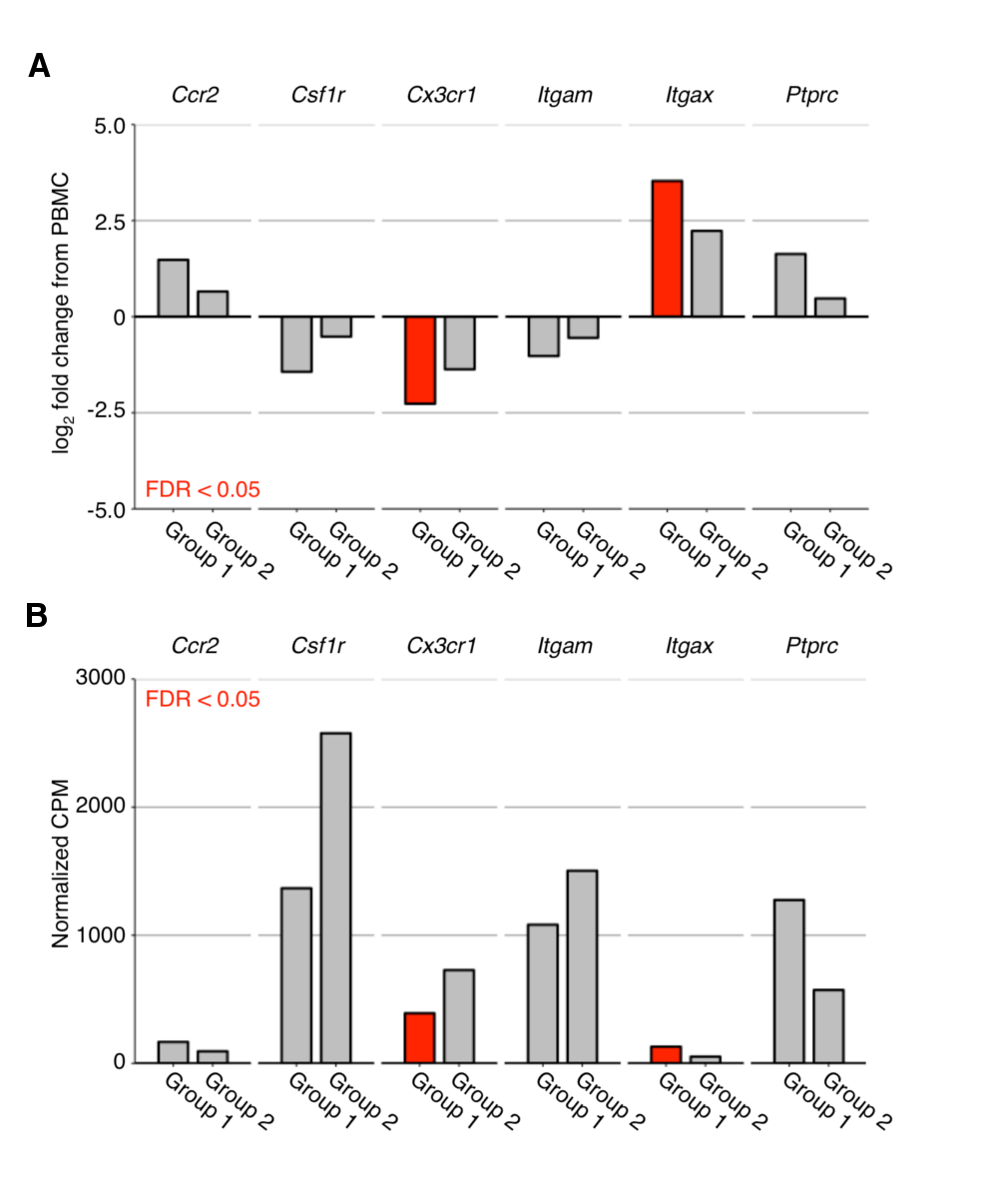

Supplement: Supplementary file 4 — Figure S4. Inflammatory monocyte markers at mRNA level. Common mouse monocyte markers at an mRNA level in ONH Monocytes vs. PBMCs by fold change from control (A) and by normalized CPM (counts per million) (B). DE genes (FDR < 0.05) are shown in red. (Itgam encodes CD11b, Itgax encodes CD11c, Ptprc encodes CD45). (TIFF 3547 kb) [file 13024_2018_303_MOESM4_ESM.tiff]

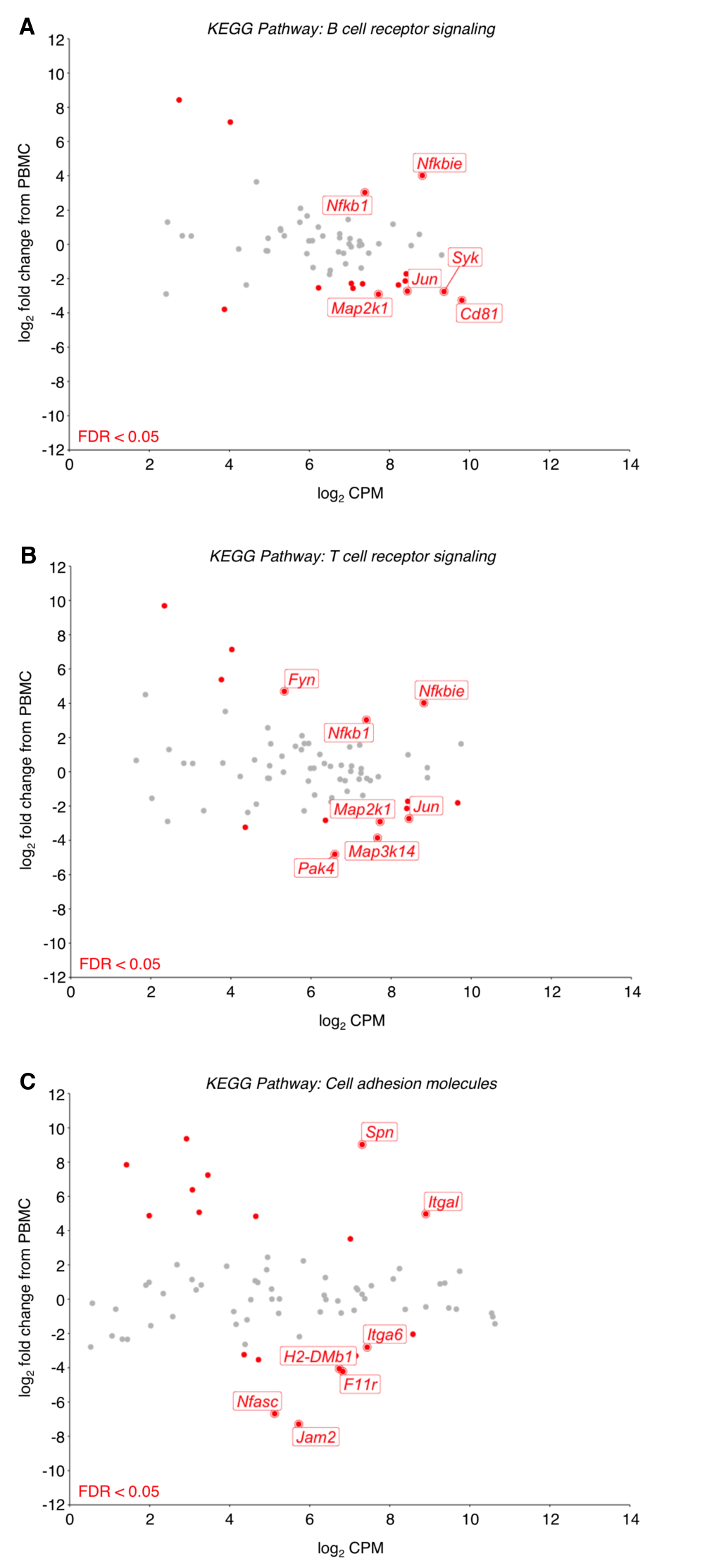

Supplement: Supplementary file 6 — Figure S5. KEGG analysis of enriched gene sets in ONH Monocytes Group 1. Scatter plots of genes by fold change from PBMCs (y) and CPM (x). (A) Genes enriched in KEGG: mmu04662, B cell receptor signalling, (B) KEGG: mmu04660, T cell receptor signalling, and (C) KEGG: mmu04514, cell adhesion molecules. Grey = not DE, red = DE (FDR < 0.05). DE genes at FDR < 0.01 with a log2 CPM > 5 are named. (TIFF 6547 kb) [file 13024_2018_303_MOESM6_ESM.tiff]

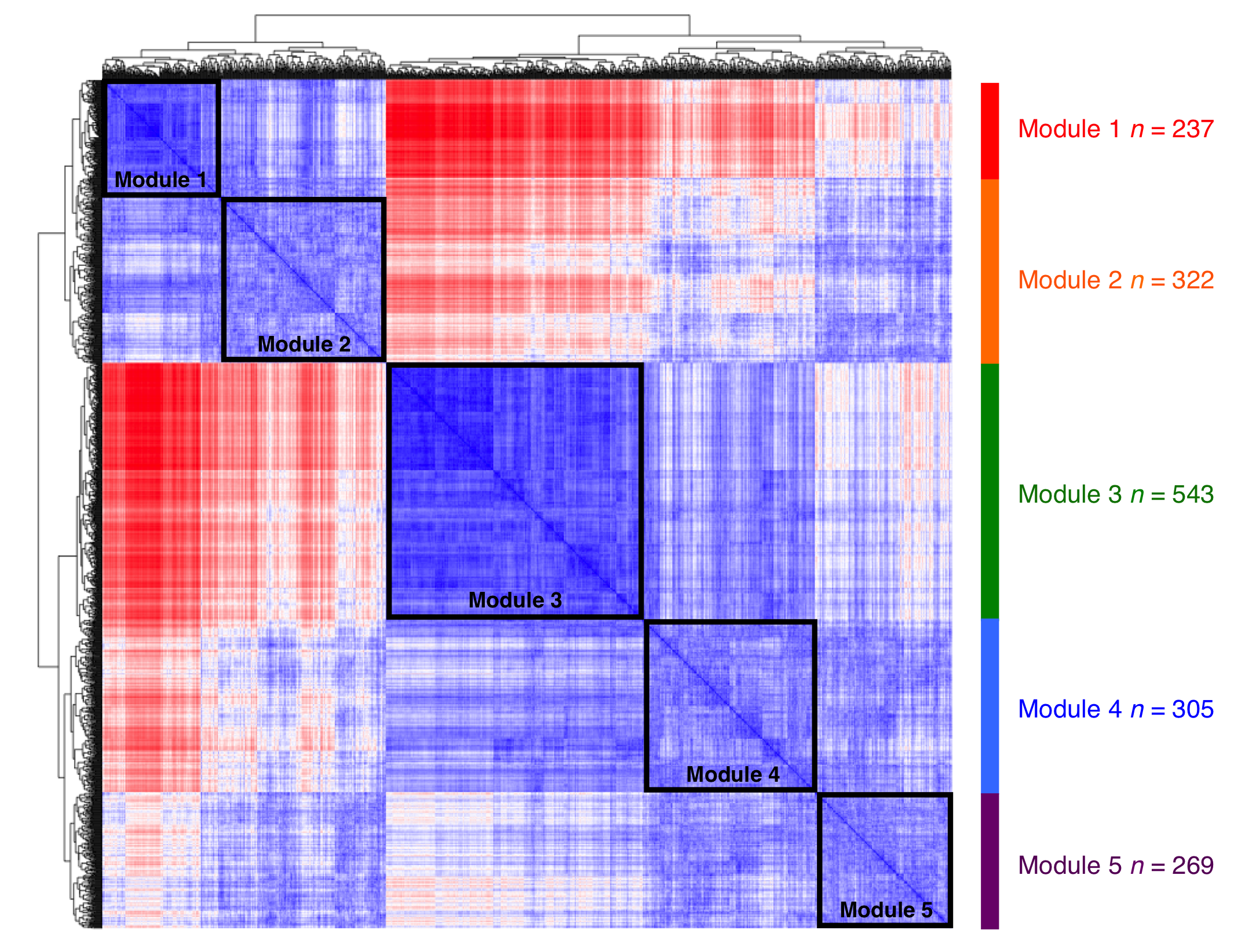

Supplement: Supplementary file 9 — Figure S6. Modular analysis of DE genes. Modules 1–5 were generated by hierarchical clustering (Spearman’s) based on DE genes (FDR < 0.05) between ONH Monocytes Group 1 vs. PBMCs. Each module is color-coded and the number of genes in the module is shown. Individual modules then underwent further analysis (see Results). (TIF 10259 kb) [file 13024_2018_303_MOESM9_ESM.tif]

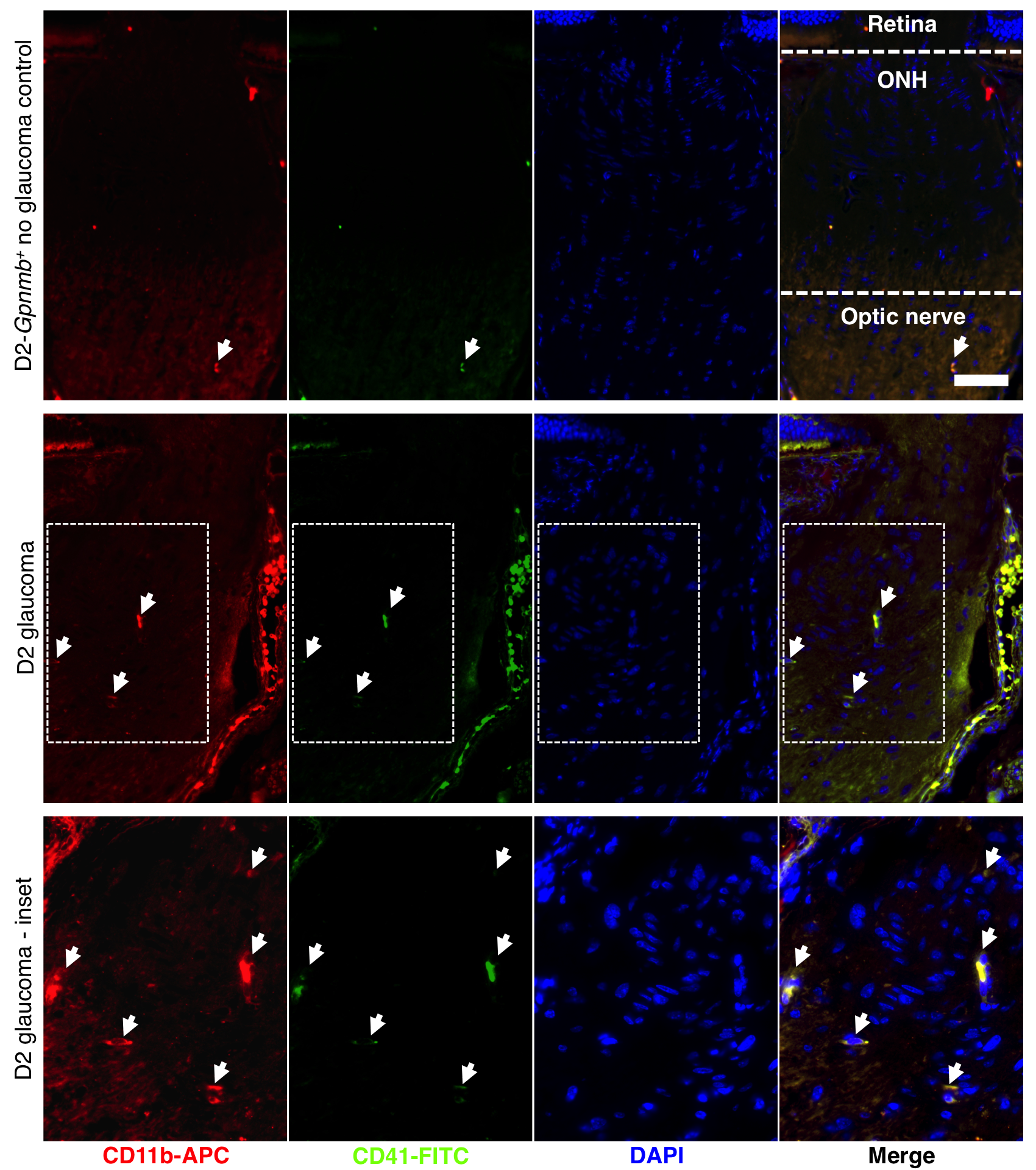

Supplement: Supplementary file 12 — Figure S7. Monocytes entering the ONH during glaucoma are platelet bound. ONHs assessed by flow cytometry demonstrate that the majority of monocytes entering the ONH during glaucoma pathogenesis are platelet bound (Fig. 3). To further confirm that monocyte-platelet aggregates were present in the ONH in glaucoma, immunofluorescence using antibodies against CD41 (a platelet marker, green) and CD11b (a monocyte maker, red) was performed in longitudinal sections of D2 and D2-Gpnmb+ ONH tissue (n = 4 / group, see upper right panel for ONH location for all upper and middle panels). The majority of monocytes that enter the ONH during glaucoma in D2 eyes are platelet-bound. APC = allophycocyanin (red), FITC = fluorescein isothiocyanate (green). Scale bar = 100 μm. (TIF 9562 kb) [file 13024_2018_303_MOESM12_ESM.tif]

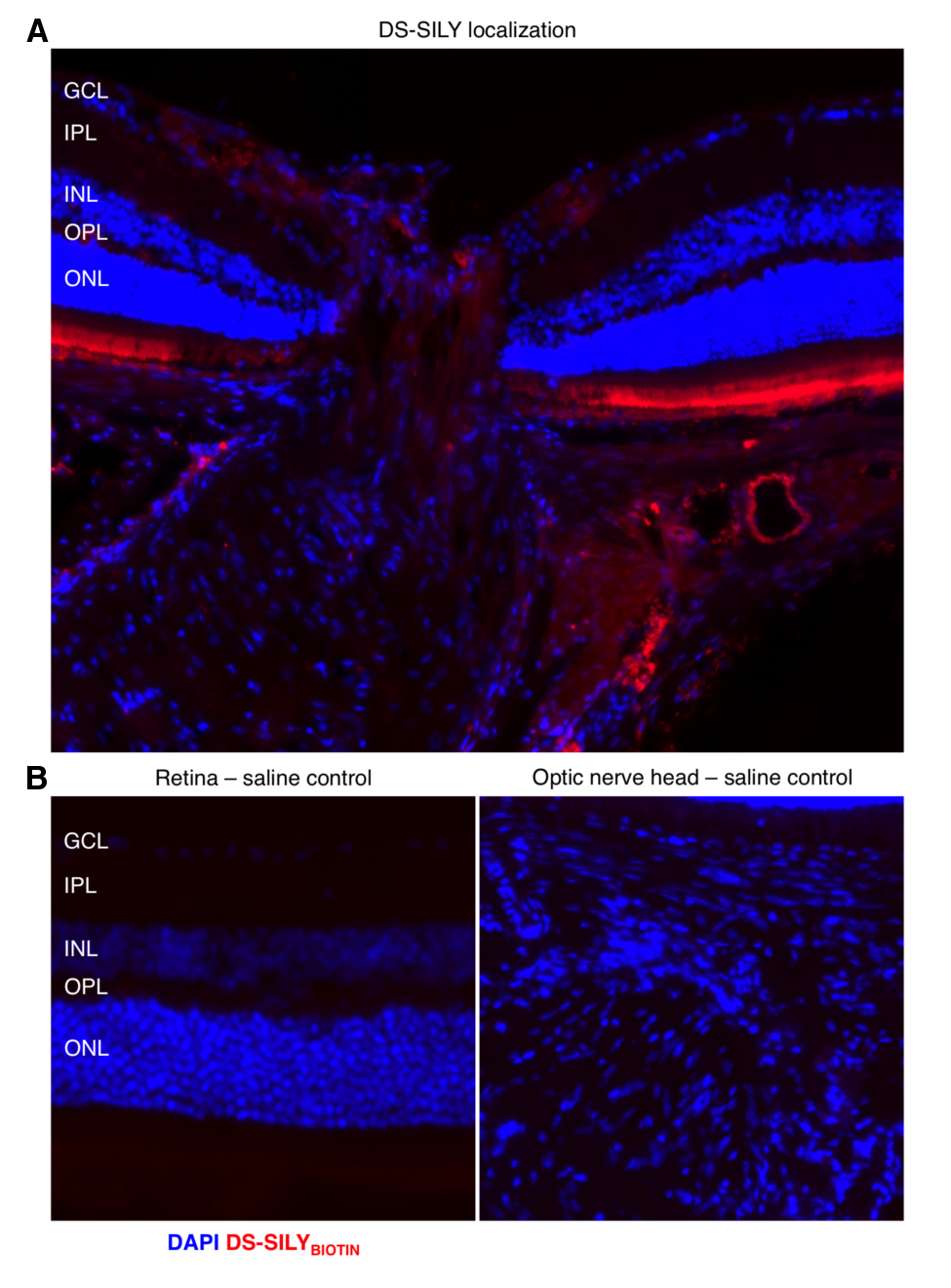

Supplement: Supplementary file 13 — Figure S8. DS-SILY binds to collagen in the retina, ONH, and surrounding vasculature. Eyes from mice that had been administered DS-SILYBIOTIN were assessed by immunofluorescence. DS-SILYBIOTIN clearly makes it to the eye and binds to inner retina vasculature (GCL, IPL), optic nerve head vasculature, and the collagen of rod outer segments (red). No fluorescence is seen in control retina and ONH (B) (n = 5 for both conditions). GCL (ganglion cell layer), IPL (inner plexiform layer), INL (inner nuclear layer), OPL (outer plexiform layer), ONL (outer nuclear layer). (TIFF 3770 kb) [file 13024_2018_303_MOESM13_ESM.tiff]

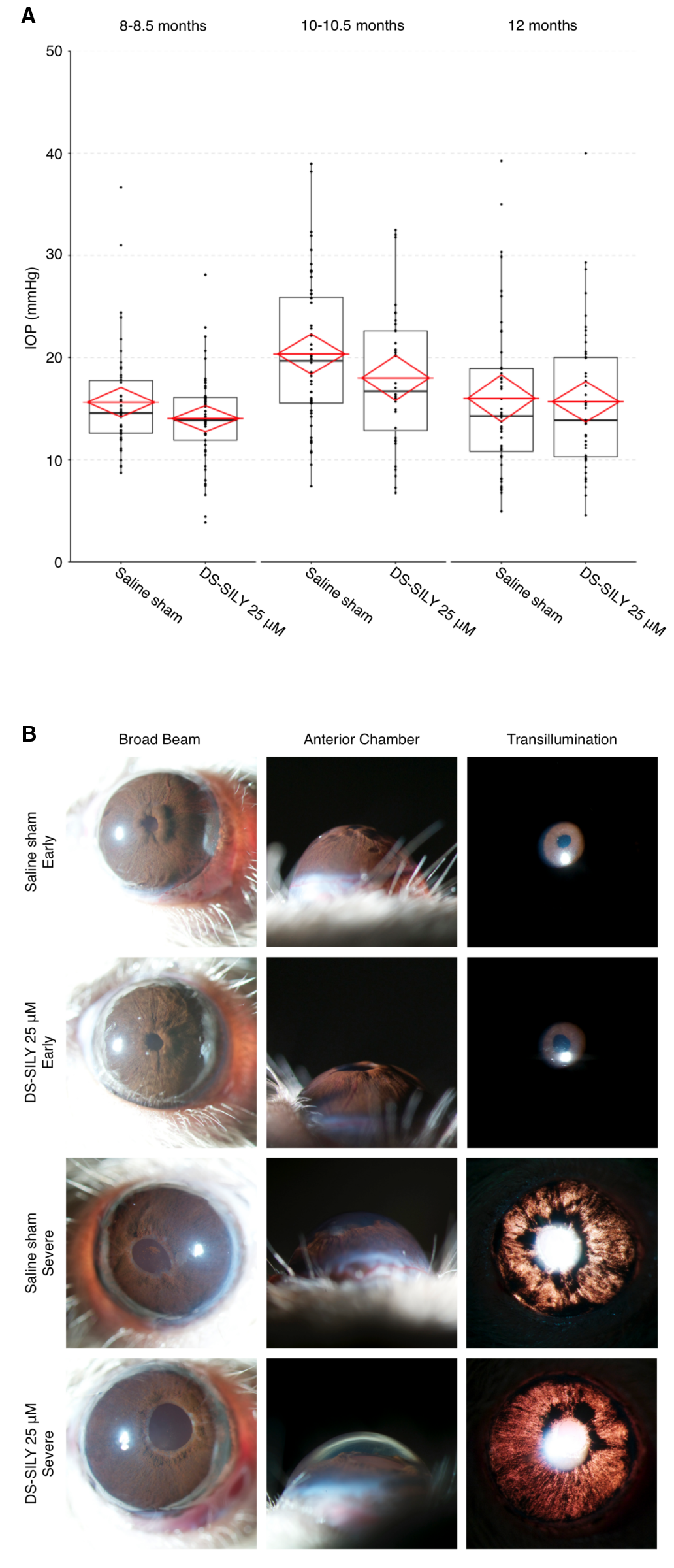

Supplement: Supplementary file 14 — Figure S9. Mice administered DS-SILY have IOP elevating anterior segment disease similar to saline sham controls. IOP profiles (A) and clinical presentation of iris disease (B) (n > 40 all conditions). IOP is not significantly different between cohorts within the same age-group. Iris disease (iris pigment dispersion resulting in asynchronous ocular hypertension) progressed at a similar rate and reached a severe state in all groups within the same time-frame. For boxplots, the upper and lower hinges represent the upper and lower quartiles. The centerline of each diamond (red) represents the mean, and the upper and lower diamond points represent 95% confidence intervals of the mean. (TIFF 7256 kb) [file 13024_2018_303_MOESM14_ESM.tiff]

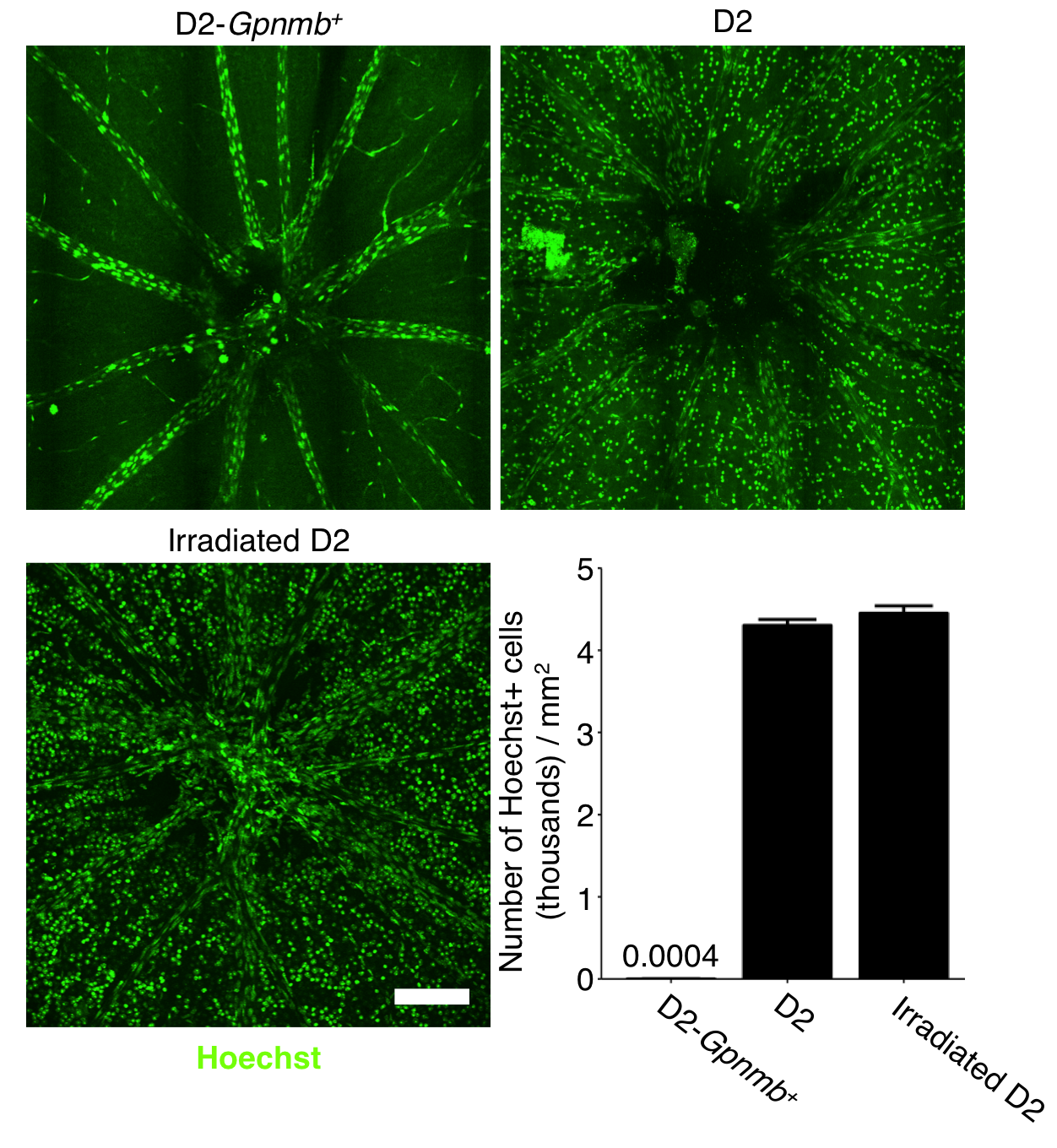

Supplement: Supplementary file 16 — Figure S10. Vascular leakage occurs early in D2 glaucoma. To explore retinal vascular damage and leakage in glaucoma, mice (9–9.5 mo of age) were intravenously injected with the DNA binding dye Hoechst. Retinas were then flat-mounted and Hoechst+ nuclei (excluding vascular endothelial cell nuclei identified by their more elliptical shape) were counted from 8 representative images of the retina. In control D2.Gpnmb+ mice Hoechst was bound only to cells within the vasculature (green, i.e. no leakage into surrounding retina). However, by 9 mo of age leakage was evident in the retinas of D2 mice (as demonstrated by nuclear labelling of all cells surrounding the retinal vasculature). Vascular leakage was evident in retinas from irradiated D2 mice that are protected from monocyte entry and glaucomatous neurodegeneration suggesting that monocyte entry in glaucoma (at least in part) must be driven by an active inflammatory process. (n = 30 D2-Gpnmb+; 18 D2; 20 irradiated D2). Scale bar = 100 μm. The number (0.0004) above D2-Gpnmb+ represents the data point. (TIF 4988 kb) [file 13024_2018_303_MOESM16_ESM.tif]

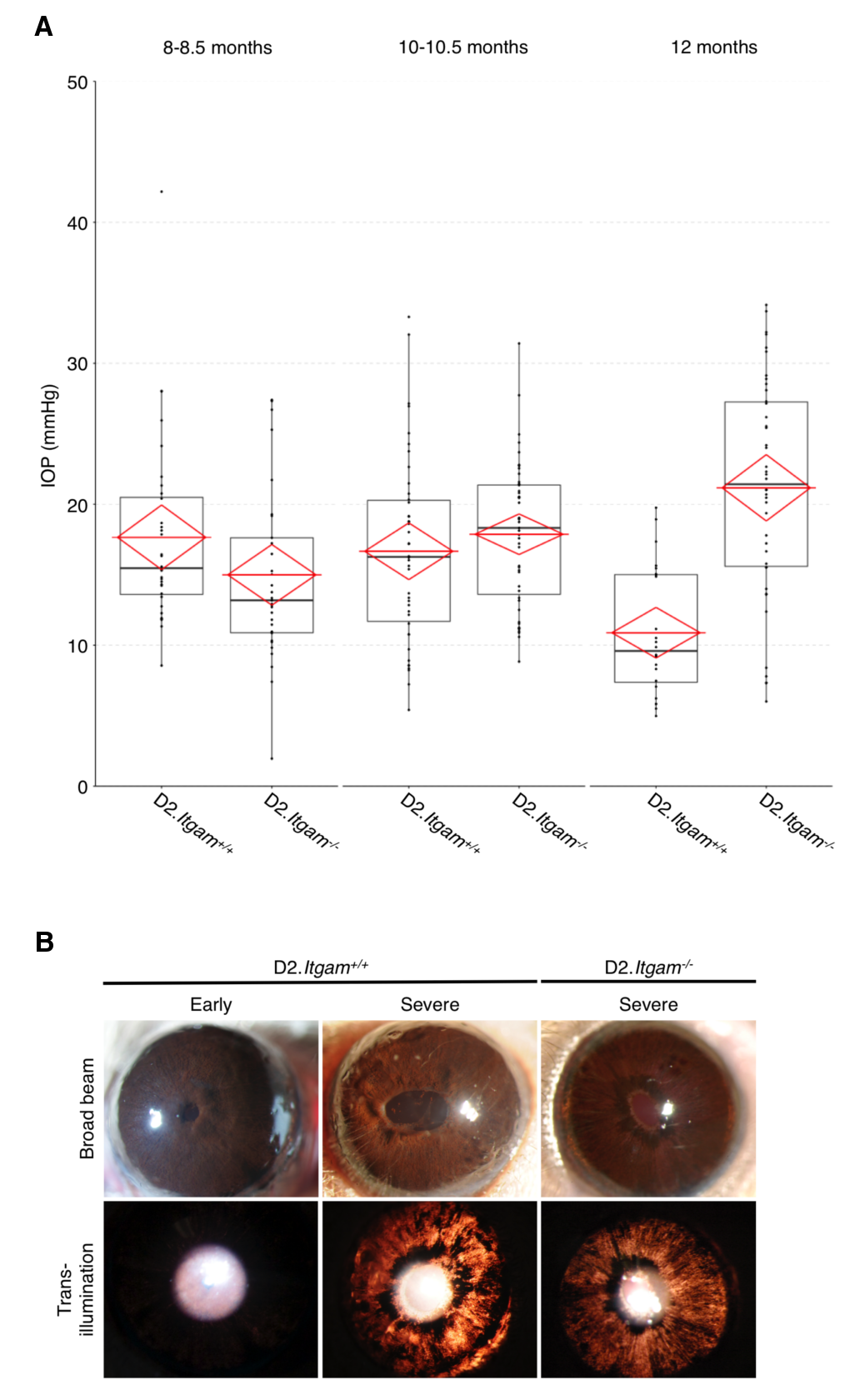

Supplement: Supplementary file 17 — Figure S11. D2.Itgam−/− mice have IOP elevating anterior segment disease similar to wild-type controls. IOP profiles (A) and clinical presentation of iris disease (B) (n > 40 all conditions). IOP is not significantly different between cohorts within the same age-group at 8 and 10 months of age, but D2.Itgam−/− eyes were more resistant to the IOP decline that usually occurs around 12 months of age (P < 0.01). Iris disease (iris pigment dispersion resulting in asynchronous ocular hypertension) progressed at a similar rate and reached a severe state in all groups within the same time-frame. For boxplots, the upper and lower hinges represent the upper and lower quartiles. The centerline of each diamond (red) represents the mean, and the upper and lower diamond points represent 95% confidence intervals of the mean. (TIFF 5109 kb) [file 13024_2018_303_MOESM17_ESM.tiff]
